# CHK1 inhibition increases the therapeutic response to radiotherapy via antitumor immunity in ARID1A-deficient colorectal cancer

**DOI:** 10.1038/s41419-025-07912-6

**Published:** 2025-08-01

**Authors:** Jhen-Yu Chen, Tao-Wei Ke, Shu-Fen Chiang, Wei-Ze Hong, Hsin-Yu Chang, Ji-An Liang, Yuan-Yao Tsai, Chi-Hsien Huang, William Tzu-Liang Chen, K. S. Clifford Chao, Kevin Chih-Yang Huang

**Affiliations:** 1 Department of Biomedical Imaging and Radiological Science, China Medical University, Taichung, Taiwan; 2 Translation Research Core, China Medical University Hospital, China Medical University, Taichung, Taiwan; 3 Department of Colorectal Surgery, China Medical University Hospital, China Medical University, Taichung, Taiwan; 4 School of Chinese Medicine and Graduate Institute of Chinese Medicine, China Medical University, Taichung, Taiwan; 5 Lab of Precision Medicine, Feng-Yuan Hospital, Ministry of Health and Welfare, Taichung, Taiwan; 6 Proton Therapy and Science Center, China Medical University Hospital, China Medical University, Taichung, Taiwan; 7 Institute of Molecular Medicine and Bioengineering, National Yang Ming Chiao Tung University, Hsinchu, Taiwan; 8 Department of Radiation Oncology, China Medical University Hospital, China Medical University, Taichung, Taiwan; 9 Department of Radiotherapy, School of Medicine, China Medical University, Taichung, Taiwan; 10 Graduate Institute of Biomedical Sciences, China Medical University, Taichung, Taiwan; 11 Department of Colorectal Surgery, China Medical University Hsinchu Hospital, China Medical University, Hsinchu, Taiwan; 12 School of Medicine, China Medical University, Taichung, Taiwan; 13 Cancer Biology and Precision Therapeutics Center, China Medical University, Taichung, Taiwan

**Keywords:** Radiotherapy, Cell death and immune response

## Abstract

Colorectal cancer (CRC) ranks among the most commonly diagnosed cancers globally and is characterized by high mortality rates and significant intertumoral heterogeneity driven by somatic mutations and an immunosuppressive tumor microenvironment (TME). Despite the success of immune checkpoint blockers (ICBs) in various malignancies, the immunosuppressive TME limits their therapeutic efficacy in the majority of CRC patients. Therefore, strategies to unleash antitumor immunity are imperative to increase the therapeutic outcomes of these patients. ARID1A mutation is frequently observed in cancers and is known to be associated with tumor activity and poor prognosis, such as colorectal cancer. Additionally, ARID1A deficiency is associated with a reduced mismatch repair capacity, increased cancer mutability, and increased infiltration of immune cells, thus potentiating the efficacy of ICBs. In this study, we revealed that ARID1A regulates CHK1 protein stability through DDB1-mediated ubiquitination. ARID1A deficiency results in CHK1 upregulation and cytosolic single-strand DNA (ssDNA) accumulation. Targeting the ATR/CHK1 axis triggers cancer cell-intrinsic innate immunity via the STING-mediated DNA-sensing pathway, thereby enhancing the therapeutic efficacy of radiotherapy (RT) and ICBs in ARID1A-deficient tumors. Taken together, these findings suggest that targeting the CHK1 checkpoint may serve as a therapeutic strategy to remodel the TME and enhance the response to radiotherapy and ICBs in ARID1A-deficient CRC.

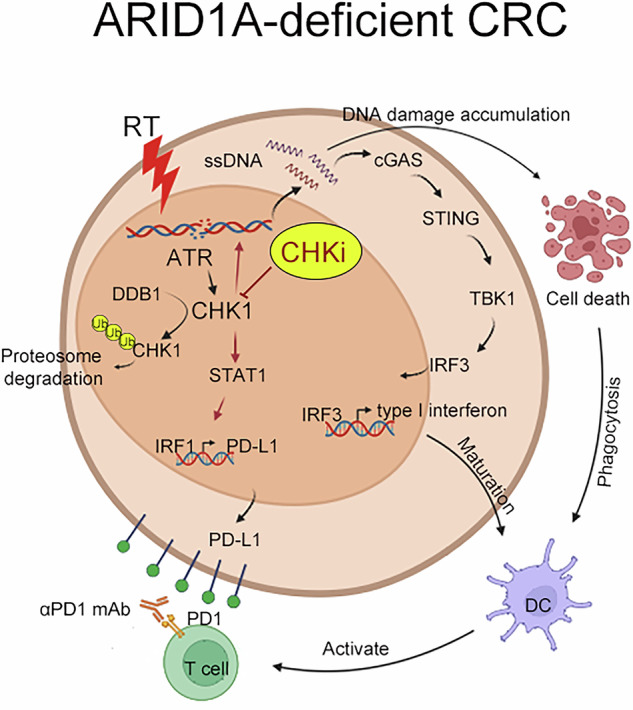

## Introduction

Colorectal cancer (CRC) is one of the most prevalent cancers worldwide [1]. Due to the intertumoral heterogeneity caused by somatic mutations and the immunosuppressive tumor microenvironment (TME), a considerable proportion of CRC patients develop local recurrence and distant metastasis within 5 years after treatment. Despite the success of immune checkpoint blockers (ICBs) in various malignancies, the immunosuppressive TME also limits their therapeutic efficacy in patients with CRC. Therefore, enhancing antitumor immunity to improve the therapeutic outcomes of patients with poorly immunologic CRC is necessary.

ARID1A is a key component of the SWI/SNF chromatin-remodeling complex, which is essential for DNA transcription, replication, and damage repair [2]. ARID1A gene mutations have been identified in approximately 10–15% of CRC cases [3, 4]. These ARID1A loss-of-function mutations resulted in reduced protein expression and impaired chromatin remodeling activity, and were strongly associated with tumor progression, distant metastases, and shorter overall survival [5–8]. Moreover, clinicopathological analyses revealed that ARID1A protein loss or reduced expression occurs in 77% of CRC patients [6, 9], and is associated with the advanced tumor-node-metastasis (TNM) stage, suggesting that ARID1A is a key tumor suppressor in CRC and its loss is strongly linked to CRC progression and metastasis. Given the high frequency of ARID1A loss or inactivation in cancer, the exploitation of anticancer therapeutics based on ARID1A status has been investigated. Recent studies have shown that targeting synthetic lethal partners of ARID1A uncovers selective vulnerabilities in ARID1A-mutant cancers [10], including inhibition of EZH2, ATR, PARP, and HDAC6. Xu et al. further showed that combining radiotherapy (RT) and ATR inhibition significantly increased the selective vulnerability of ARID1A-deficient CRC [11]. These studies demonstrated that synthetic lethal targeting of ARID1A is a promising approach for the development of novel cancer-targeted therapies. Additionally, ARID1A deficiency is associated with a reduced mismatch repair (MMR) capacity and increased cancer mutability to recruit infiltrating immune cells, increasing the therapeutic efficacy of ICBs [12, 13]. ARID1A-deficient cancer cells exhibit increased type I interferons (IFN) response signaling, which promotes CD8^+^ T-cell recruitment and cytolytic activity [14]. However, ARID1A deficiency also regulated programmed cell death ligand 1 (PD-L1) expression through PI3K/AKT signaling to modulate the immune microenvironment [15, 16], suggesting that additional therapeutic strategies are needed for ARID1A-deficient tumors.

Checkpoint kinase 1 (CHK1) plays a critical role in cell cycle regulation and serves as a key regulatory protein of ATR-mediated DNA damage [17]. CHK1 inhibition disrupts the ability of cells to repair DNA damage, resulting in the accumulation of DNA damage and ultimately leading to cancer cell death. Recently, several studies reported that targeting CHK1 significantly affects the TME by increasing cytotoxic T-cell infiltration to reshape antitumor immunity [18, 19]. CHK1 inhibition contributes to the accumulation of cytosolic DNA, triggering the cGAS-STING signaling pathway to enhance antitumor responses by production of type I IFNs [20]. Previous studies indicated that inhibition of the ATM/CHK2 axis promotes cGAS/STING signaling to enhance antitumor immunity in ARID1A-deficient tumors, revealing that targeting DNA damage checkpoints may be a therapeutic strategy for ARID1A-deficient tumors [21].

In this study, we revealed that ARID1A regulates CHK1 protein stability by DDB1-mediated ubiquitination. ARID1A deficiency leads to CHK1 upregulation and results in cytosolic ssDNA accumulation. Here, we demonstrated that targeting the ATR/CHK1 axis triggers cancer cell-intrinsic immunogenicity by the STING-mediated DNA-sensing pathway to enhance the therapeutic efficacy of radiotherapy and ICBs in ARID1A-deficient tumors. Taken together, these results indicate that targeting CHK1 may represent a promising therapeutic strategy to remodel the TME and enhance the responsiveness of ARID1A-deficient tumors to radiotherapy and ICBs.

## Materials and methods

### Mice

Six-week-old female BALB/c mice were purchased from Biolasco Taiwan Co., Ltd. All day-to-day care was provided by trained staff members in the mouse house. All procedures were approved by the local animal care committee (CMUIACUC-2018-182). All procedures involving the use and care of animals were performed according to the Guide for the Care and Use of Laboratory Animals published by the National Research Council.

### Cell culture

CT26 (BALB/c mouse undifferentiated colon carcinoma, CRL-2638, ATCC), SW620 (CCL-227^TM^), HCT116 (CCL-247^TM^), HT29 (HTB-38), and 293T (human embryonic kidney, CRL-3216, ATCC) cells were used in this study. The cell lines were cultured in complete RPMI 1640 medium (Gibco #31800022, Thermo Fisher Scientific) supplemented with 10% fetal bovine serum (Gibco #16000044, Thermo Fisher Scientific), 2 mM L-glutamine (Gibco #25030081, Thermo Fisher Scientific), and 1% (v/v) penicillin/streptomycin (Gibco #10378016, Thermo Fisher Scientific) and maintained at 37 °C in 5% CO_2_. They were not further authenticated but were cultured for a limited number of passages (<10 passages). The cell lines were tested for mycoplasma contamination using PCR.

### Antibodies and Western blot analysis

The antibodies used in this study included the following: anti-cleaved caspase-3 (#9661, Cell Signaling Technology and IR96-401, iReal Biotech.), anti-cleaved PARP (#5625, Cell Signaling), anti-GAPDH (IR3-8, iReal Biotech.), anti-p-TBK1 (AP1026, ABclonal), anti-TBK1 (A2573, ABclonal), anti-ARID1A (#12354, Cell Signaling Technology), anti-CHK1 (A7653, ABclonal and 25887-1-AP, Proteintech), anti-p-CHK1 (AP0578, ABclonal), anti-γ-H2AX (#2577, Cell Signaling), and anti-PD-L1 (ab205921, Abcam and AP17952-1, Proteintech). All secondary antibodies (HRP-conjugated anti-rabbit and anti-mouse antibodies) were obtained from Santa Cruz Biotechnology.

Total lysates (30 μg) were separated on an SDS–PAGE gel and subsequently transferred to PVDF membranes (Millipore, MA, USA) [22] for immunoblot analyses with the indicated antibodies overnight at 4 °C. The membranes were subsequently probed with HRP-conjugated secondary antibodies for 1 h at room temperature. All the antibodies were diluted in T-Pro Protein Free Blocking Buffer (BioLion Tech., Taipei, Taiwan). The membranes were then treated with Immobilon Western Chemiluminescent HRP Substrate (Millipore, CA, USA), visualized using an ImageQuant™ LAS 4000 biomolecular imager (GE Healthcare, Amersham, UK), processed using Adobe Photoshop and quantified using ImageJ software (NIH, MD, USA). Each blot was stripped with immunoblotting stripping buffer (BioLion Tech.) before a subsequent incubation with other antibodies to ensure an accurate analysis [23].

### RT-qPCR

Total RNA was extracted from the cell lines with TRIzol (Invitrogen, CA, USA), quantified by measuring the absorbance at 260 nm, and then reverse transcribed into cDNA using iScript™ Reverse Transcription Supermix (Bio-Rad, CA, USA) according to the manufacturer’s instructions [24, 25]. Primers were designed using the Primer Design Tool (NCBI, USA) based on sequence information from the NCBI database. RT-qPCR was conducted in a final reaction volume of 20 μL with iQ™ SYBR® Green Supermix (Bio-Rad, CA, USA) using a CFX96 Touch Real-Time PCR Detection System (Bio-Rad). Each sample was analyzed in triplicate, and GAPDH was used as a reference gene for normalization. Relative gene expression levels were calculated using the 2^−ΔΔCt^ method, and comparisons between gene expression levels were performed using *t* tests.

### Evaluation of the immune cell profiles induced by CHK1 inhibitor and local RT in vivo

BALB/c mice (female, 6 weeks old) were housed according to the institutional guidelines approved by the China Medical University Institutional Animal Care and Use Committee. Briefly, CT26 cells (2.5 × 10^5^ cells/mouse) were suspended in 100 μL of 30% Matrigel and subcutaneously inoculated into the right leg of each mouse. After 7 days, a CHK1i (CCT244747, 10 mg/kg/mouse, intraperitoneal injection) was administered, and local radiotherapy (5 Gy) was administered. The tumor volume was measured every 3 days throughout the study. The longest and shortest diameters (*L* and *W*, respectively) of the tumors were measured using Vernier calipers every 3 days, and the tumor volume (*V*) was calculated with the formula *V* = (*L* × *W*^2^)/2. At the end of the experiment, the mice were sacrificed, and tumor tissues were collected and subjected to immunofluorescence staining.

### Immunofluorescent staining

The antibodies used in this study were as follows: anti-CD3 (ab11089, Abcam) and anti-mouse CD8 (ab217344, Abcam). The tissue sections on slides were deparaffinized and subjected to heat-mediated antigen retrieval with an antigen unmasking solution (H3300, Vector Laboratories, Burlingame, CA). The tissue sections on the slides were incubated with 5% horse serum for 10 min. Tissue sections (3 µm thick) were stained with the indicated primary antibodies and FITC-conjugated secondary antibodies and counterstained with DAPI [26, 27].

Staining for immune cells was positive when detected in the tumor-infiltrating lymphocytes (TILs) and was evaluated using a microscope (OLYMPUS BX53, Tokyo, Japan). For the detection of TILs, the tissue was viewed at ×40 magnification, and the area with the highest density of CD3^+^ and CD8^+^ TILs within the malignant cells was counted at ×400 magnification (no. of TILs/high-power field). The average number of tumor-infiltrating immune cells in five high-power fields was included in the evaluation [28–30].

### Co-culture assay

The human monocytic leukemia cell line THP-1 was cultured and maintained in RPMI 1640 medium supplemented with 10% FBS, 2 mM glutamine, 1 mM sodium pyruvate, and 1% P/S. THP-1 cells were differentiated into immature DCs (iDC) by treatment with 1500 IU/mL rhIL-4 (Sino Biological, Beijing, China) and 1500 IU/mL rhGM-CSF (Sino Biological, Beijing, China) in culture medium for at least 7 days [31]. The cytokine-supplemented culture medium was changed every 2–3 days. HCT116 and HT29 cells were each irradiated with 5 Gy and incubated for 24 h, then cocultured with THP-1 iDCs for an additional 24 h in six-well plates using Transwell inserts with 0.4-µm pores. The phenotype of the THP-1 iDCs was then analyzed via RT-qPCR [32].

### Combinational therapies of CHKi local radiotherapy and anti-PD1 blockade in vivo

A total of 2.5 × 10^5^ CT26 cells in 100 μL of 30% Matrigel were inoculated into the right flanks of BALB/c mice. The treatments were initiated on Day 7 after tumor cell inoculation: CHK1i (intraperitoneal injection, 10 mg/kg/mouse 4 times). On Day 14, the mice received local radiotherapy (5 Gy), and anti-mouse PD1 antibodies were administered on Days 13, 16, and 19 (5 mg/kg, intraperitoneal injection, 3 times with 3-day intervals between injections; Bio×Cell clone RMP1-14, NH, USA). The longest and shortest diameters (*L* and *W*, respectively) of the tumors were measured using Vernier calipers (Sata, Shanghai, China) every 3 days, and the tumor volume (*V*) was calculated using the following formula: *V* = (*L* × *W*^2^)/2. At the end of the experiment, the mice were sacrificed, and tumor tissues were collected for lysis and flow cytometry and subjected to immunofluorescence staining and RT-qPCR [33].

### Flow cytometry analysis of immune cell profiles

Tumors were dissected from the mice, weighed and then placed in Petri dishes containing blank RPMI media at room temperature to prevent dehydration [34–36]. The tumors were minced into small pieces (1–2 mm) with a beaver blade, filtered through a 70 μm strainer, centrifuged, and then resuspended in blank RPMI media. Thereafter, the cell suspensions were layered over Ficoll-Paque media and centrifuged at 1025×*g* for 20 min. The layer of mononuclear cells was transferred to a conical tube, 20 mL of complete RPMI media was added, the mixture was gently mixed, and the sample was centrifuged at 650 × *g* for 10 min twice. Finally, the supernatant was removed, and the TILs were resuspended in complete RPMI media.

Then, the TILs were resuspended in 500 μL of staining buffer (2% BSA and 0.1% NaN_3_ in PBS). The cells were stained with different surface marker panels: (1) DC cells: CD11c-PE Cy7 (N418), MHC II-spark blue 550 (M5/114.15.2), CD86-BV421 (GL-1), and CD80-BV510 (16-10A1) are from BioLegend. (2) CD4/CD8 T cells: CD3-pacific blue (17A2), CD45-BV785 (30-F11), CD4-BV570 (RM4-5), and CD8a-BB515 (53-6.7) are from BioLegend. (3) Regulatory T cells: CD45-BV785 (30-F11), CD4-BV570 (RM4-5), CD25-V605 (PC61), and CD127-PE (A7R34) are from BioLegend. For intracellular staining, TILs were fixed and permeabilized with staining buffer after cell surface staining. The cells were then stained with TNFα-BV421(MP6-XT22), IFNγ-PE(XMG1.2), and IL2-PE Cy7 (JES6-5H4) for 45 min. The samples were washed twice with Perm Wash Buffer and analyzed with Cytek® Northern Lights/Aurora Spectral flow cytometer (Cytek Biosciences Inc., CA, USA).

### Tissue microarray (TMA) construction for immunohistochemistry

Patients who were diagnosed with colorectal cancer and treated between 2011 and 2014 at China Medical University Hospital were enrolled in our cohort [37–39]. The TMA included resected primary tumor tissue and corresponding normal mucosal samples, and tissue collection was approved by the Institutional Review Board (IRB) of China Medical University Hospital [Protocol number: CMUH107-REC2-008].

IHC staining was performed on 3-μm-thick TMA sections with the following indicated antibodies: anti-human ARID1A (#12354, Cell Signaling), CD3 (ab11089, Abcam), CD8 (ab4055, Abcam), p-CHK1 phospho-S345 (ab47318, Abcam), ssDNA (MAB3299, MERCK), and PD-L1 (ab205921, Abcam). This step was followed by an incubation with the HRP-conjugated avidin biotin complex (ABC) kit (Vector Laboratories, CA, USA) and the HRP substrate DAB chromogen (Vector Laboratories) and counterstaining with hematoxylin [29, 40].

ARID1A, p-CHK1, ssDNA, and PD-L1 levels in the tumor tissues were evaluated and scored based on the intensity and percentage of cells positive using the histoscore (*H* score), which was calculated by performing a semiquantitative assessment of both the intensity of the staining (0: negative staining; 1: weak; 2: moderate; and 3: strong staining) and the percentage of immunopositive cells. The H score ranged from 0 to 300, and we categorized the data into low or high groups based on the average *H* score [22, 25, 30].

### Statistical analysis

All the experiments were conducted at least 3 times. All the statistical analyses were performed using GraphPad Prism 9 statistical software (GraphPad Software, CA, USA) [40]. The data were analyzed using two-way ANOVA followed by Bonferroni’s post hoc correction, one-way ANOVA followed by Dunnett’s post hoc test, or an unpaired *t*-test, where appropriate. The data are presented as the means ± SEMs. Student’s *t*-test was used to compare the differences in tumor sizes and positive cell counts between the two groups. ANOVA was used for comparisons of the results involving combinations of CCT244747, RT, and PD1 blockade among the groups. *p* < 0.05 was considered to indicate a significant difference. The survival period was defined as the time from surgery to cancer-specific death, and overall survival (OS) was assessed by the Kaplan‒Meier survival analysis.

## Results

### ARID1A deficiency is negatively correlated with CHK1 protein expression

**Fig. 1:**
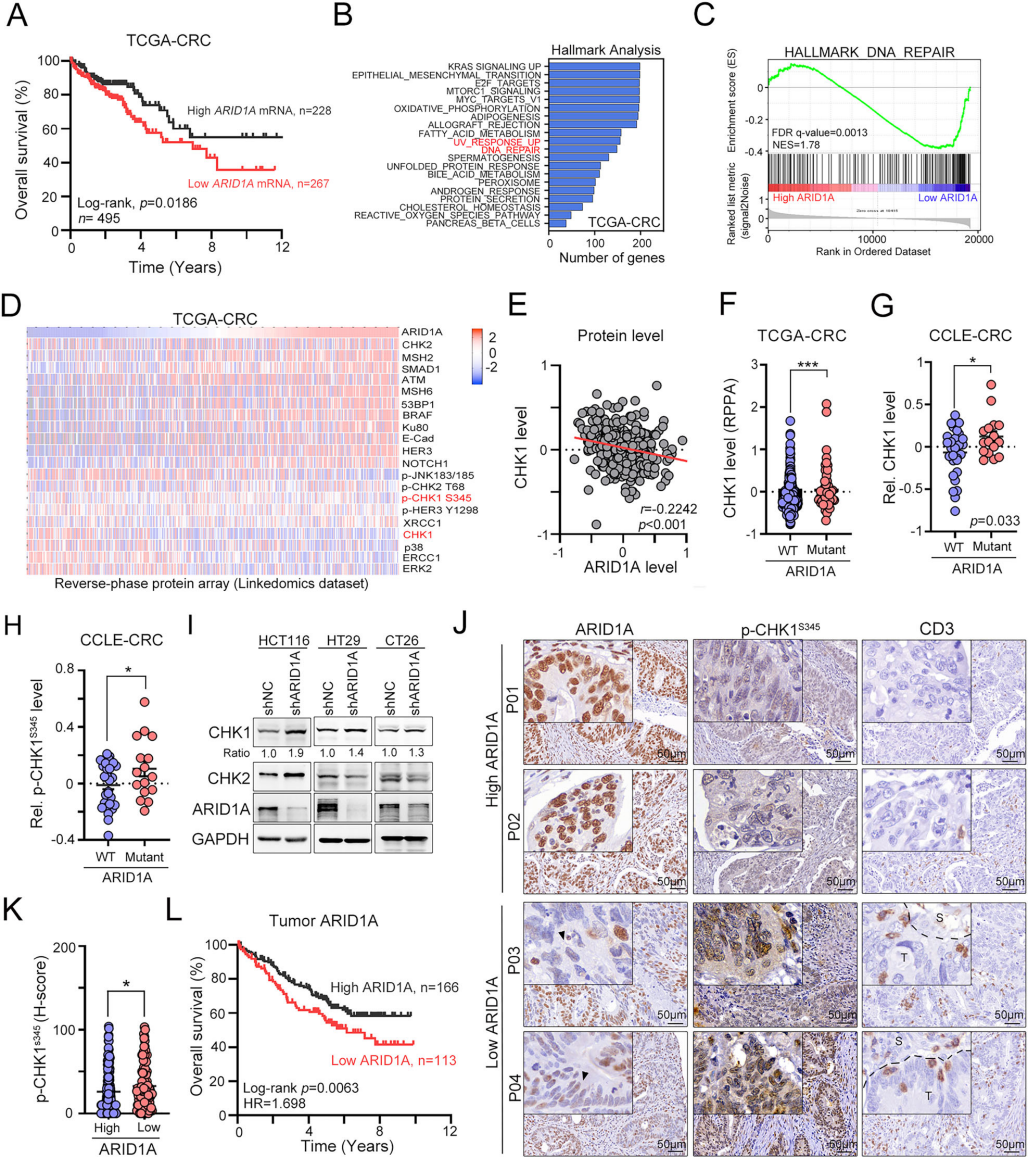
CHK1 signaling was enhanced in CRC tumors with mutant or low ARID1A. High tumor *ARID1A* mRNA expression was associated with favorable OS in colorectal cancer patients in the TCGA dataset (*n* = 495, Log-rank *p* = 0.0186). **A** GSEA analysis on the CRC patients stratified by *ARID1A* mRNA level. The significant hallmark gene signature was shown (*p* < 0.05). **B** The GSEA analysis on the HALLMARK_DNA_REPAIR signature between ARID1A-high and ARID1A-low subgroups. **C** Heatmap representing expression profiles of most differentially expressed proteins between ARID1A-high and ARID1A-low CRC patients from the TCGA dataset (*p* < 0.05). **D** The relationship between tumor ARID1A and CHK1 protein level was examined in colorectal cancer patients in the TCGA dataset (*r* = 0.2242, *n* = 491, *p* < 0.001). **E** The CHK1 protein levels in ARID1A-WT and ARID1A-Mut CRC patients from the TCGA dataset were analyzed (*p* < 0.001, *n* = 464). **F** CHK1 protein levels in ARID1A-WT and ARID1A-Mut CRC cells from the CCLE dataset were analyzed (*p* < 0.001, *n* = 54). **G** The p-CHK1^S345^ protein levels in ARID1A-WT and ARID1A-Mut CRC cells from the CCLE dataset (*p* < 0.001, *n* = 54). **H** HCT116, HT29, and CT26 cells were infected with lentivirus carrying shRNA against ARID1A. Cells were harvested for Western blot analysis. **I** The representative images of tumor ARID1A, p-CHK1^S345^, and tumor-infiltrating CD3^+^ T cells in colorectal cancer patients (*n* = 279). **J** The phospho-CHK1 expression in ARID1A-High and ARID1A-Low CRC patients (*p* < 0.001, *n* = 279). **K** CRC patients with high tumor ARID1A protein expression were associated with favorable OS (*n* = 279, Log-rank *p* = 0.0063).

First, we examine the overall survival in the ARID1A-mutated CRC patients in the CRC database retrieved from The Cancer Genome Atlas Program (TCGA-CRC, *n* = 522). We found that there was no significant difference in overall survival between ARID1A-mutated and ARID1A-WT CRC patients in the TCGA-CRC dataset (*p* = 0.578, Fig. S1A). Previous studies indicated that ARID1A protein loss or reduced expression occurs in 77% of CRC patients [6, 9], which might be due to its promoter hypermethylation and mutation [41]. Therefore, we stratified these CRC patients based on the *ARID1A* mRNA level (*n* = 495, Fig. 1A). Patients with low *ARID1A* mRNA were correlated with shorter overall survival in CRC patients (*p* = 0.0186, Fig. 1A). Additionally, *ARID1A* mRNA expression was also lower in tumor tissue compared to normal tissue in CRC (Fig. S1B). We then systematically investigated signaling pathways by gene set enrichment analysis (GSEA) of *ARID1A* mRNA levels in the TCGA-CRC dataset to identify which gene signatures might be targeted to increase the therapeutic efficacy of radiotherapy in *ARID1A*-low CRC. We found that the hallmark DNA_REPAIR signature and UV response were significantly changed and increased, respectively, in *ARID1A*-low CRC patients (Figs. 1B, C and S1C), which was consistent with previous studies [10, 42]. We then analyzed reverse-phase protein array (RPPA) data in the TCGA-CRC database (*n* = 491), which included 10.8% ARID1A-mutated and 89.2% ARID1A-WT tumors. The average *Z* scores were 0.2512 in ARID1A-WT tumors and −0.1095 in ARID1A-mutated tumors, indicating significantly lower ARID1A protein levels in ARID1A-mutated CRC patients. As shown in Fig. 1D, we identified a subset of proteins involved in diverse molecular pathways whose expression levels differed significantly between tumors with high versus low ARID1A protein expression, such as DNA damage checkpoint kinase CHK1 and its active form (phospho-CHK1^S345^). Both total CHK1 and phospho-CHK1^S345^ expression levels were significantly higher in ARID1A-low CRC patients, compared to ARID1A-high patients (Fig. 1D). These CHK1 protein levels were inversely correlated with ARID1A protein expression (Fig. 1E). Furthermore, CHK1 protein levels were also significantly higher in ARID1A-mutated CRC patients (TCGA dataset, Fig. 1F, *p* < 0.001) and CRC cell lines (CCLE dataset, Fig. 1G, *p* = 0.033). Phospho-CHK1^S345^ levels were also significantly elevated in ARID1A-mutated CRC cell lines (Fig. 1H, *p* < 0.05), suggesting that the activated DNA damage response in ARID1A-mutated CRC tumors. To confirm this correlation, we generated ARID1A-silenced CRC cell lines, which were characterized as ARID1A-WT. Following ARID1A knockdown, we observed a notable increase in CHK1 protein levels across all three cell lines, as shown in Fig. 1I. These results indicated that the ARID1A loss contributes to CHK1 upregulation.

To further demonstrate the clinical relevance between ARID1A and CHK1 signaling, we analyzed the expression of ARID1A and p-CHK1^S345^ by immunohistochemical analysis (*n* = 279, Fig. 1J). We then stratified CRC patients into two subgroups based on the tumor ARID1A expression. We found that patients with low ARID1A expression presented increased levels of p-CHK1^S345^ within the tumor tissue (Fig. 1J and K, *p* < 0.05). Low ARID1A expression significantly correlated with poorer survival outcomes in CRC patients (Fig. 1L), which was consistent with the results from *ARID1A* mRNA (Fig. 1A). We then analyzed the association between clinic parameters and ARID1A expression in CRC patients (Table S1). ARID1A expression was significantly correlated with MMR status (*p* < 0.05, Table S1). We also found a high density of tumor-infiltrating CD3^+^ T cells in ARID1A-low CRC patients (Fig. S1D). Similarly, higher expression of *CD8A*, *GZM.B*, and *IFNγ1* mRNA in ARID1A-mutated CRC patients compared to ARID1A-WT CRC patients in the TCGA-CRC dataset (Fig. S1E–G). Collectively, these results indicated that CHK1 may represent a potential therapeutic target for ARID1A-low CRC patients. By targeting CHK1, it may be possible to enhance the efficacy of treatment strategies in this specific subset of CRC patients, potentially improving outcomes and addressing an unmet clinical need.

### Targeting CHK1 significantly sensitizes ARID1A-deficient CRC to radiotherapy

**Fig. 2:**
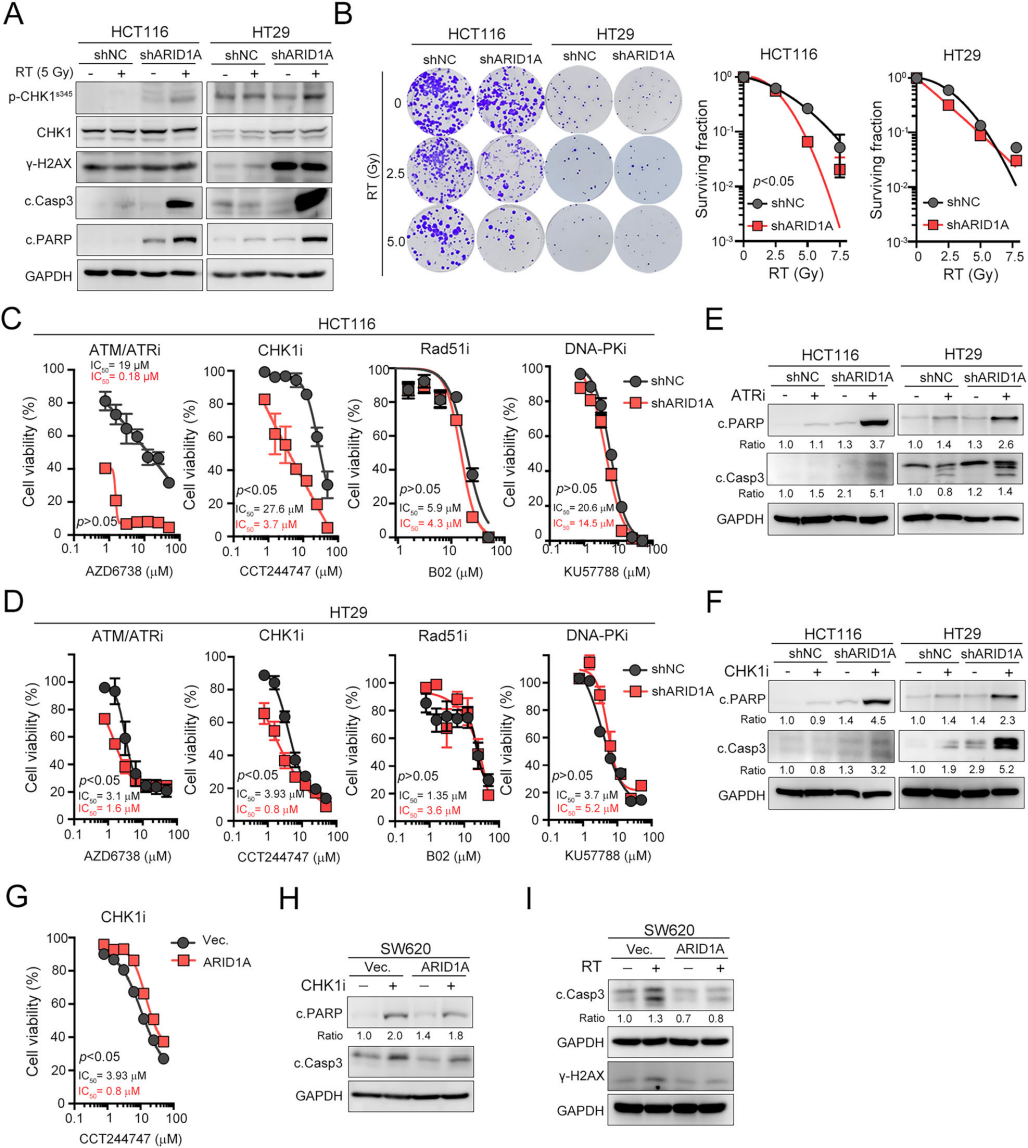
Inhibition of ATR/CHK1 signaling significantly promoted cell death in ARID1A-deficient CRC. **A** HCT116^shNC^ and HCT116^shARID1A^ cell was irradiated (5 Gy) and harvested after 24 h. The apoptotic cell marker caspase-3 and PARP were examined by Western blot. The same experiment was carried out in HT29^shNC^ and HT29^shARID1A^ cells. **B** HCT116^shNC^ and HCT116^shARID1A^ cells were irradiated (0, 2.5, and 5 Gy) and harvested after 7 days (*n* = 3). The same experiment was carried out in HT29^shNC^ and HT29^shARID1A^ cells (*n* = 3, *p* < 0.05). **C** HCT116^shNC^ and HCT116^shARID1A^ cells were treated with different small molecules, AZD6738 (ATR inhibitor), CCT244747 (CHK1 inhibitor), B02 (RAD51 inhibitor), and KU57788 (DNA-PK inhibitor) for 72 h. Cell viability was examined by CCK8 assay (*n* = 3). **D** HT29^shNC^ and HT29^shARID1A^ cells were treated with different small molecules, AZD6738 (ATR inhibitor), CCT244747 (CHK1 inhibitor), B02 (RAD51 inhibitor), and KU57788 (DNA-PK inhibitor) for 72 h. Cell viability was examined by CCK8 assay (*n* = 3). **E** HCT116^shNC^ and HCT116^shARID1A^ cells were treated with ATR inhibitor AZD6738 (10 μM) for 24 h. Cells were harvested for Western blot analysis. The same experiment was carried out in HT29^shNC^ and HT29^shARID1A^ cells (*n* = 3). **F** HCT116^shNC^ and HCT116^shARID1A^ cells were treated with CHK1 inhibitor CCT244747 (10 μM) for 24 h. Cells were harvested for Western blot analysis. The same experiment was carried out in HT29^shNC^ and HT29^shARID1A^ cells (*n* = 3). **G** SW620 cells were overexpressed HA-Vector and HA-ARID1A for 24 h, and then treated with CHK1 inhibitor CCT244747 for an additional 48 h. Cell viability was examined by CCK8 assay (*n* = 3). **H** SW620 cells were overexpressed HA-Vector and HA-ARID1A for 24 h, and then treated with CHK1 inhibitor CCT244747 (10 μM) for an additional 24 h. Cells were harvested for Western blot analysis. These data were obtained from three independent experiments, and the values represent the means ± SEM. One-Way ANOVA *t*-test. *p < 0.05, **p < 0.01 and ***p < 0.001. **I** SW620 cells were overexpressed HA-Vector and HA-ARID1A for 24 h, and then irradiated (5 Gy). Cells were harvested for western blot analysis in post-RT 24 h (*n* = 3).

The loss of ARID1A has been reported to disturb the balance of DNA repair efficacy and impair the G2/M DNA damage checkpoint [21], thereby increasing susceptibility to specific genotoxic treatments, such as PARP inhibitors and radiotherapy [42]. Therefore, we generated ARID1A-silenced CRC cell lines based on the expression of endogenous ARID1A (Fig. S2) and evaluated the response to RT (Fig. 2A). As shown in Fig. 2A, irradiation triggered significant γ-H2AX induction and enhanced apoptotic cell death in ARID1A-silenced cells. The level of cleaved caspase-3 and its downstream PARP proteins, the hallmarks of apoptosis, were significantly increased in ARID1A-silenced cells after RT treatment. Moreover, the level of p-CHK1^S345^ was also significantly increased in ARID1A-silenced cells. Additionally, the surviving colonies were significantly decreased in ARID1A-silenced HCT116 and HT29 cells after RT treatment, especially in HCT116 cells (Fig. 2B), indicating that ARID1A knockdown increased the sensitivity to RT.

We then employed specific small-molecule inhibitors that target different DNA repair proteins, such as ATR (ceralasertib/AZD6738), CHK1 (CCT244747), RAD51 (B02) and DNA-PK (KU57788) to assess the cell viability in ARID1A-silenced cells [43–45]. As shown in Fig. 2C and D, inhibition of ATR and CHK1 markedly reduced the cell viability in ARID1A-silenced HCT116 and HT29 cells. However, inhibition of RAD51 and DNA-PK did not decrease the cell viability in ARID1A-silenced cells compared to control cells. These results suggested that targeting ATR/CHK1-mediated homogenous recombination (HR) DNA repair mechanism might significantly induced cell death in ARID1A-deficient CRC cells. Indeed, the levels of cleaved caspase-3 and PARP were markedly increased in ARID1A-deficient CRC cells following ATR or CHK1 inhibitor treatment (Fig. 2E and F). In contrast, overexpression of ARID1A decreased the response of these cells to CHK1 inhibition (Fig. 2G and H) and RT (Fig. 2I). Taken together, these results indicated that CHK1 inhibition results in pronounced cell death in ARID1A-deficient CRC tumors.

**Fig. 3:**
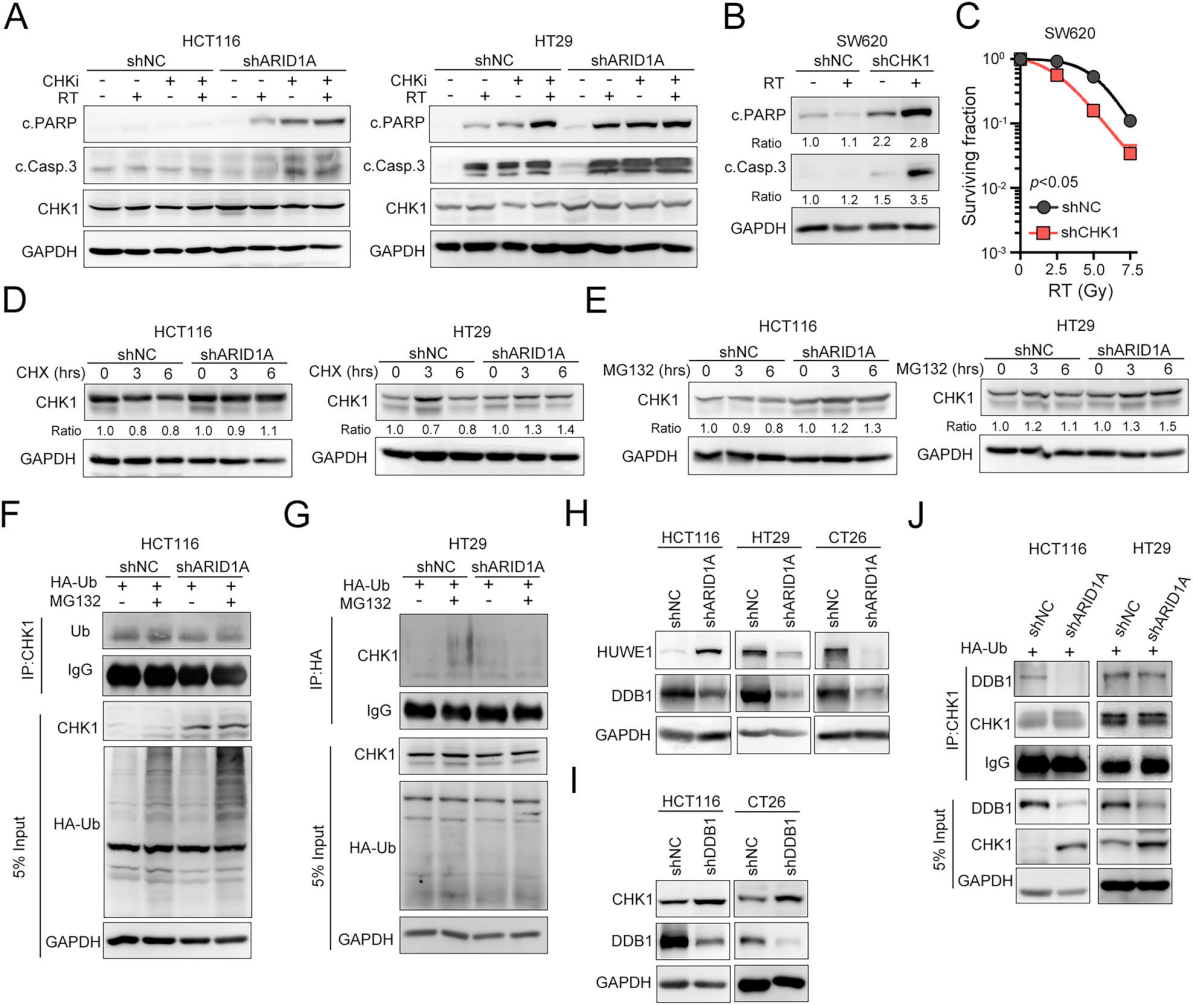
ARID1A downregulates CHK1 by DDB1-mediated proteasome degradation. **A** HCT116^shNC^ and HCT116^shARID1A^ cells were treated with CHK1 inhibitor CCT244747 (10 μM) and RT (5 Gy) for 24 h. Cells were harvested for Western blot analysis. The same experiment was carried out in HT29^shNC^ and HT29^shARID1A^ cells (*n* = 3). **B** SW620^shNC^ and SW620^shCHK1^ cells were irradiated (5 Gy) for 24 h. Cells were harvested for Western blot analysis. **C** SW620^shNC^ and SW620^shCHK1^ cells were irradiated (0, 2.5, 5, and 7.5 Gy) for 7 days. Survival fraction was calculated based on the plating efficacy. **D** HCT116^shNC^ and HCT116^shARID1A^ cells were treated with CHX (100 μg/mL) for different periods and harvested for western blot analysis. The same experiment was carried out in HT29^shNC^ and HT29^shARID1A^ cells (*n* = 3). **E** HCT116^shNC^ and HCT116^shARID1A^ cells were treated with MG132 (10 μM) for different periods and harvested for western blot analysis. The same experiment was carried out in HT29^shNC^ and HT29^shARID1A^ cells (*n* = 3). **F** HCT116^shNC^ and HCT116^shARID1A^ cells were transfected with HA-Vector and HA-ubiquitin (Ub) for 42 h and treated with MG132 (10 μM) for an additional 6 h. Cells were harvested for immunoprecipitation and western blot analysis. **G** HT29^shNC^ and HT29^shARID1A^ cells were transfected with HA-Vector and HA-Ub for 42 h and treated with MG132 (10 μM) for an additional 6 h. Cells were harvested for immunoprecipitation and western blot analysis. **H** HCT116, HT29, and CT26 cells were infected with lentivirus carrying shRNA against ARID1A. Cells were harvested for Western blot analysis. **I** HCT116, HT29, and CT26 cells were infected with lentivirus carrying shRNA against DDB1. Cells were harvested for Western blot analysis. **J** HCT116^shNC^ and HCT116^shARID1A^ cells were transfected with HA-Vector and HA-Ub for 48 h. Cells were harvested for immunoprecipitation and western blot analysis. These data were obtained from three independent experiments, and the values represent the means ± SEM. One-Way ANOVA *t*-test. *p < 0.05, **p < 0.01 and ***p < 0.001.

We then evaluate the synergistic effect of CHK1 inhibition and RT treatment (Fig. 3A). As depicted in Fig. 3A, the combination treatment with the CHK1 inhibitor and RT markedly increased the level of cleaved caspase-3 and PARP in two ARID1A-silenced cell lines. Furthermore, knockdown of CHK1 in SW620 cells, which exhibit low endogenous ARID1A levels, significantly enhanced sensitivity to RT, as evidenced by increased levels of cleaved caspase-3 and PARP, and a marked reduction in cell viability (Fig. 3B and C). These results suggested that irradiation exacerbates DNA damage, which cannot be effectively repaired in ARID1A-deficient CRC cells, thereby enhancing downstream ATR/CHK1 signaling activation. Targeting CHK1 with a small-molecule inhibitor significantly blocks cell cycle arrest for DNA repair, resulting in cell death.

### ARID1A mediates CHK1 downregulation through DDB1-dependent proteasomal degradation

We then investigated the detailed mechanism by which ARID1A regulates CHK1 protein expression. First, ARID1A-silenced CRC cells were treated with cycloheximide, a protein synthesis inhibitor. As shown in Fig. 3D, CHK1 expression was sustained in two ARID1A-silenced cell lines, suggesting that CHK1 was stabilized in ARID1A-deficient cells. Next, we treated cells with the proteasomal degradation inhibitor MG132. We observed that the CHK1 protein significantly accumulated in ARID1A-silenced CRC cells but not in control cells (Fig. 3E). These results suggested that ARID1A modulated CHK1 expression through a proteasomal degradation mechanism. We then overexpressed the ubiquitin protein and immunoprecipitated CHK1 to further confirm these results (Fig. 3F and G). We found that a large amount of ubiquitin was conjugated to the CHK1 protein after MG132 treatment in control cells but not in ARID1A-silenced cells (Fig. 3F and G), suggesting that ARID1A downregulates CHK1 through the ubiquitin–proteasome pathway.

Previous studies reported that two E3 ligases, DDB1 and HUWE-1, are involved in CHK1 protein stability [46–48]. Additionally, we observed a positive correlation between DDB1, HUWE-1, and ARID1A expression in the TCGA-COAD dataset (Fig. S3A and B). We examined the expression levels of DDB1 and HUWE-1 in ARID1A-silenced cell lines to investigate this possibility further. As shown in Fig. 3H, DDB1 expression was decreased in three ARID1A-silenced CRC cell lines, HCT116, HT29 and CT26. However, the level of HUWE-1 was not consistent in three ARID1A-silenced CRC cell lines. Therefore, we generated DDB1-silenced cells using a lentivirus carrying an shRNA against DDB1 (Fig. 3I). We observed increased CHK1 expression upon DDB1 silencing. Furthermore, the immunoprecipitation results showed a reduced interaction between DDB1 and CHK1 in ARID1A-silenced CRC cells (Fig. 3J). These results suggested that ARID1A downregulates CHK1 expression via DDB1-dependent ubiquitination and degradation pathway.

### Radiation-induced ssDNA elicits immune response-related gene transcription through the cGAS/STING signaling pathway

**Fig. 4:**
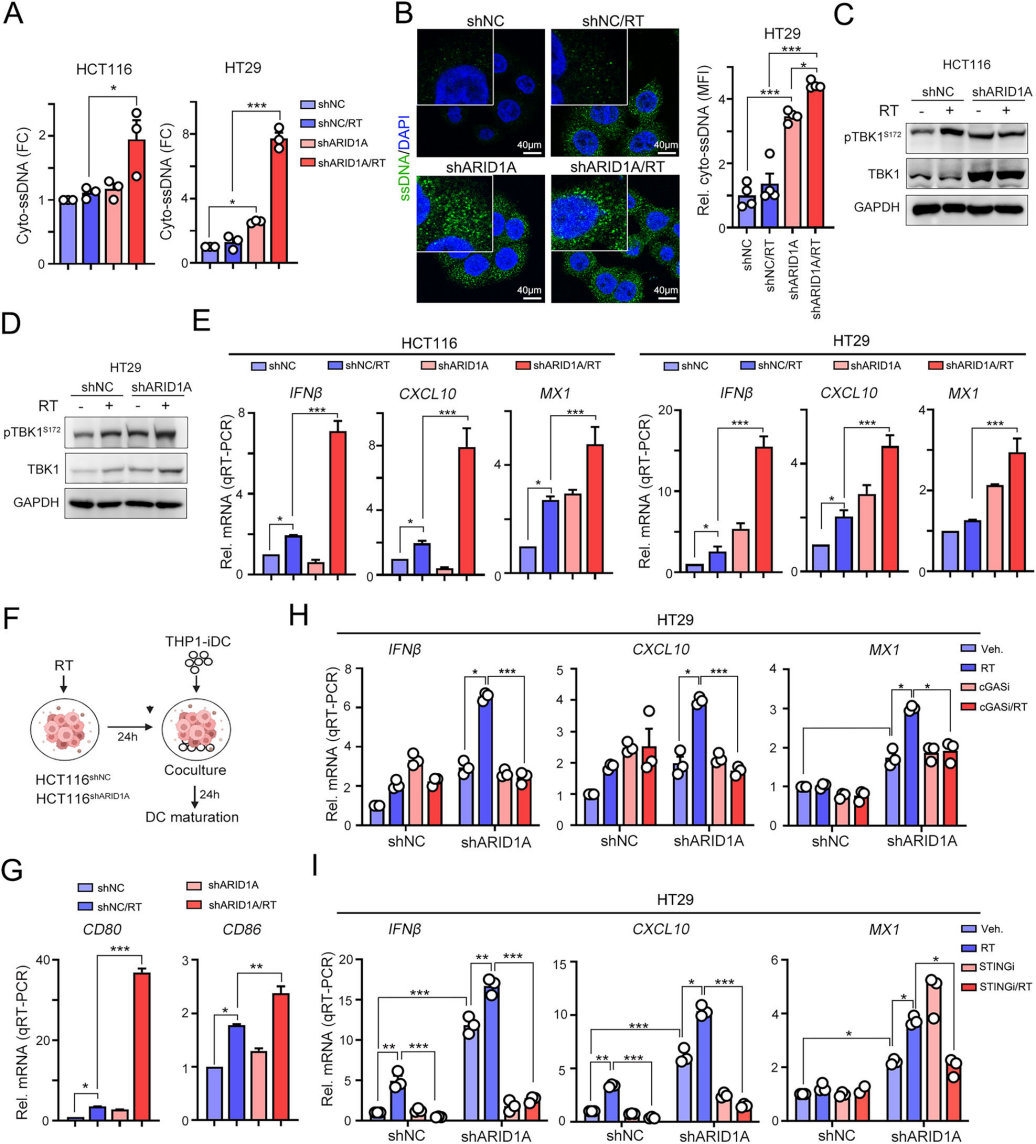
CHK1 inhibition remarkably increased RT-induced type I IFN production by ssDNA/cGAS/STING axis in ARID1A-deficient CRC cells. **A** HCT116^shNC^ and HCT116^shARID1A^ cells was treated with RT (5 Gy) for 24 h. The cytosolic fraction was isolated for single-stranded DNA analysis (*n* = 3). The same experiment was carried out in HT29^shNC^ and HT29^shARID1A^ cells (*n* = 3). **B** HT29^shNC^ and HT29^shARID1A^ cells were treated with RT (5 Gy) for 24 h. The cytosolic ssDNA was detected by specific ssDNA antibodies and analyzed by confocal microscope (*n* = 3). **C** HCT116^shNC^ and HCT116^shARID1A^ cells were treated with RT (5 Gy) for 24 h. Cells were harvested for Western blot analysis. **D** HT29^shNC^ and HT29^shARID1A^ cells were treated with RT (5 Gy) for 24 h. Cells were harvested for Western blot analysis. **E** HCT116^shNC^ and HCT116^shARID1A^ cells were treated with RT (5 Gy) for 24 h. The level of type I IFN-related genes was measured by RT-qPCR (*n* = 3). The same experiment was carried out in HT29^shNC^ and HT29^shARID1A^ cells (*n* = 3). **F** The schematic diagram of immature DC cocultured with RT-treatment HCT116 cells. **G** HCT116 cells treated with RT (5 Gy) were co-cultured for 24 h with THP1-iDCs, which had been differentiated using IL-4 (1500 IU/mL) and GM-CSF (1500 IU/mL) for 7 days. The mRNA level of DC markers (*CD86* and *CD80*) was determined by RT-qPCR(*n* = 3). **H** HT29^shNC^ and HT29^shARID1A^ cells were treated with RT (5 Gy) and cGAS inhibitor G140 (1 μM) for 24 h. The level of type I IFN-related genes was measured by RT-qPCR (*n* = 3). **I** HT29^shNC^ and HT29^shARID1A^ cells were treated with RT (5 Gy) and STING inhibitor H151 (1 μM) for 24 h. The level of type I IFN-related genes was measured by RT-qPCR (*n* = 3).

The phosphorylation of CHK1 by ATR occurs in response to ssDNA generated by replication arrest [49] or DNA-damaging agents such as RT. Both cytosolic dsDNA and ssDNA can be recognized by the cGAS/STING signaling pathway, which activates downstream type I IFN production via TBK1 and IRF3 [20]. Therefore, we first examined the level of cytosolic ssDNA in ARID1A-silenced cells in response to RT. We observed a substantial increase in cyto-ssDNA levels in ARID1A-silenced cells following RT (Fig. 4A). These results were further supported by immunofluorescence staining (Fig. 4B). The phosphorylation of TBK1 was significantly increased in ARID1A-silenced cell lines following RT (Fig. 4C and D). Furthermore, the expression of type I IFN-related genes was notably increased in ARID1A-silenced cells after RT treatment (Fig. 4E).

Type I IFNs play pivotal roles in orchestrating immune responses, particularly through their regulation of DC maturation. We hypothesized that the cancer immunogenicity of ARID1A-deficient cells was increased for dendritic cell recognition to attract infiltrating immune cells via the ssDNA/CHK1/cGAS/STING pathway. To test this, we individually irradiated HCT116^shNC^ and HCT116^shARID1A^ cells and then cocultured them with THP1-iDC cells for 24 h. Indeed, we found that the expression of DC maturation markers *CD80* and *CD86* was significantly upregulated after coculture with irradiated HCT116^shARID1A^ cells (Fig. 4F and G), suggesting that the cancer immunogenicity of ARID1A-deficient cells was strongly triggered by RT to promote DC maturation and the antitumor immune response.

**Fig. 5:**
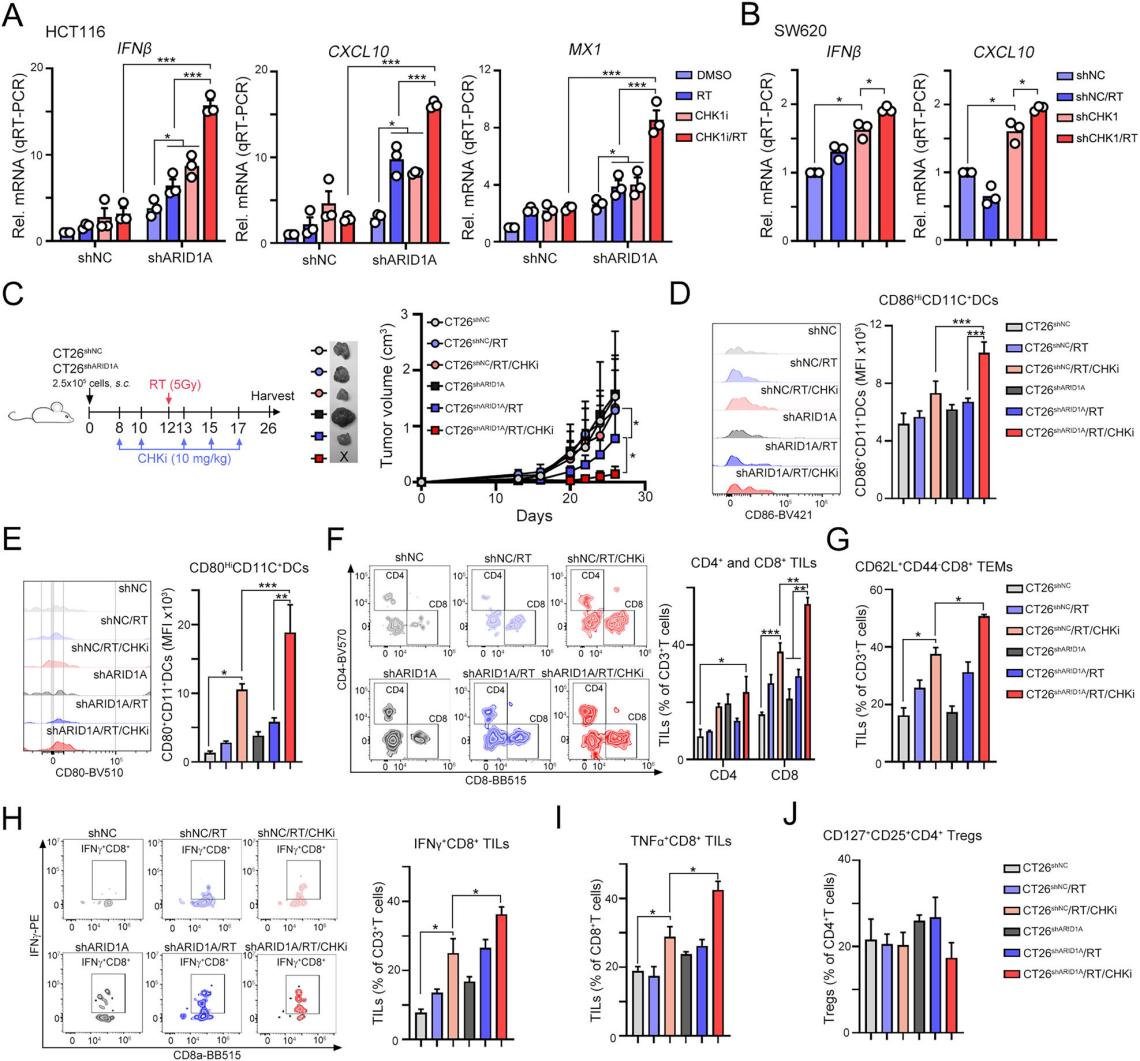
CHK1 inhibition augmented RT-induced antitumor immunity to eradicate cancer cells in vivo. **A** HCT116^shNC^ and HCT116^shARID1A^ cells were treated with RT (5 Gy) and CHK1 inhibitor CCT244747 (10 μM) for 24 h. The level of type I IFN-related genes was measured by RT-qPCR (*n* = 3). **B** SW620^shNC^ and SW620^shCHK1^ cells were irradiated (5 Gy) for 24 h. The mRNA level of *IFNβ* and *CXCL10* was measured by RT-qPCR (*n* = 3). These data were obtained from three independent experiments, and the values represent the means ± SEM. One-Way ANOVA *t*-test. *p < 0.05, **p < 0.01 and ***p < 0.001. **C** Tumor growth of CT26^shNC^ and CT26^shARID1A^-driven colon carcinoma established in BALC/c mice (*n* = 6/group) that were treated with RT (5 Gy for 1 fraction) and CHK1 inhibitor (CCT244747, 10 mg/kg) on the indicated day. Tumor growth is reported as the mean tumor volume ± SD. *p < 0.05 and **p < 0.01. CR: complete response. Two-way ANOVA *t*-test. **D** The frequency of tumor-infiltrating CD86^Hi^ CD11c^+^ DCs in resected tumors was analyzed by flow cytometry (*n* = 3–5). **E** The frequency of tumor-infiltrating CD80^Hi^ CD11c^+^ DCs in resected tumors was analyzed by flow cytometry (*n* = 3–5). **F** The density of tumor-infiltrating CD4^+^ and CD8^+^ T cells in resected tumors was analyzed by flow cytometry (*n* = 3–5). **G** The density of tumor-infiltrating CD62L^+^CD44^-^CD8^+^ TEM cells in resected tumors was analyzed by flow cytometry (*n* = 3–5). *p < 0.05. One-Way ANOVA *t*-test. **H** The density of tumor-infiltrating IFNγ^+^CD8^+^ T cells in resected tumors was analyzed by flow cytometry (*n* = 3–5). *p < 0.05. One-Way ANOVA *t*-test. **I** The density of tumor-infiltrating TNFα^+^CD8^+^ T cells in resected tumors was analyzed by flow cytometry (*n* = 3–5). *p < 0.05. One-Way ANOVA *t*-test. **J** The density of tumor-infiltrating CD127^+^CD25^+^CD4^+^ Tregs cells in resected tumors was analyzed by flow cytometry (*n* = 3–5). *p < 0.05 and ***p < 0.001. One-Way ANOVA *t*-test.

The radiation-induced upregulation of type I IFN-related gene expression was subsequently attenuated by the addition of a cGAS/STING inhibitor in ARID1A-silenced HT29 cells (Fig. 4H and I). Moreover, the levels of type I IFN-related genes were markedly increased in ARID1A-silenced cells treated with the combination of the CHK1 inhibitor and RT (Figs. 5A and S4A). Similarly, knockdown of CHK1 in SW620 cells significantly attenuated RT-induced type I IFN production (Fig. 5B). Taken together, these results indicate that RT-induced type I IFN production was mainly dependent on the ssDNA/CHK1/cGAS/STING signaling pathway in ARID1A-deficient CRC cells.

### Combination treatment with irradiation and a CHK1 inhibitor in ARID1A-deficient cells reshapes the tumor microenvironment in vivo

Since the expression of the DC maturation markers *CD80* and *CD86* mRNA was higher in ARID1A-silenced CRC cells following RT treatment, and the combination of RT with a CHK1 inhibitor further increased type I IFN-related gene expression in ARID1A-silenced CRC cells, we next evaluated whether targeting CHK1 can improve antitumor immune responses in vivo. We inoculated CT26^shNC^ and CT26^shARID1A^ cells into the right legs of immunocompetent BALB/c mice to evaluate the therapeutic efficacy of RT in combination with CHK1 inhibition. These mice were then subjected to local radiotherapy and treatment with the CHK1 inhibitor CCT244747 (intraperitoneal injection) to evaluate the therapeutic efficacy (Fig. 5C). Notably, local radiotherapy alone significantly reduced the tumor size and delayed tumor growth in the CT26^shARID1A^ subgroup compared with the CT26^shNC^ subgroup. Moreover, the combination of local radiation and the CHK1 inhibitor CCT244747 further enhanced the therapeutic efficacy in the CT26^shARID1A^ subgroup. This combined treatment approach resulted in 33% complete tumor regression (2/6) in the CT26^shARID1A^ subgroup.

**Fig. 6:**
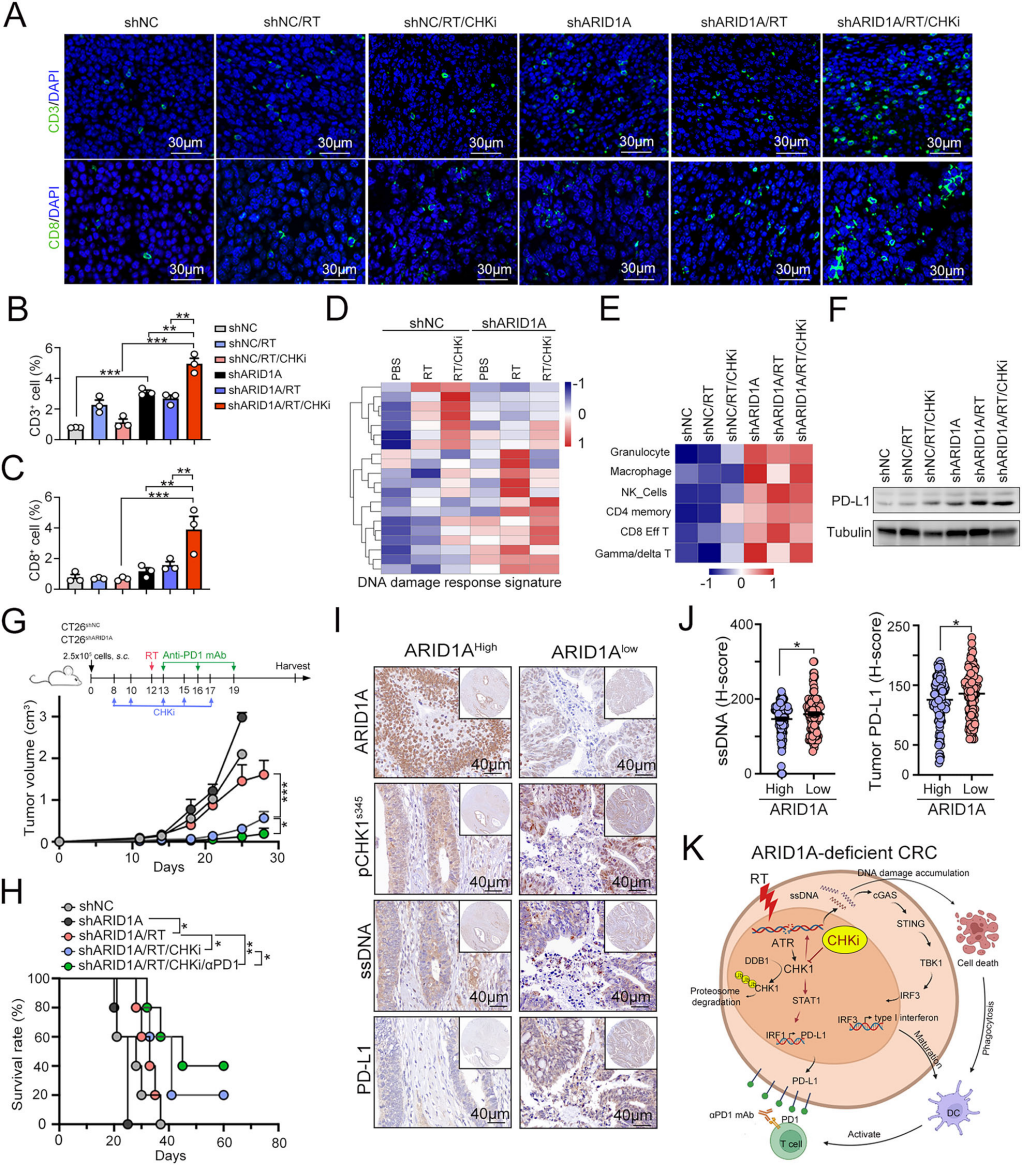
Combinational treatment with the CHK1 inhibitor and RT increased the therapeutic response to ICBs in ARID1A-deficient CRC cells. **A** The representative results of infiltration of CD3^+^ and CD8^+^ immune cells were analyzed by immunofluorescent (*n* = 3). **B** The density of CD3^+^ T cells was quantified under high-power-field microscopy (*n* = 3). These data were obtained from three independent experiments, and the values represent the means ± SEM. One-Way ANOVA *t*-test. *p < 0.05, **p < 0.01 and ***p < 0.001. **C** The density of CD8^+^ T cells was quantified under high-power-field microscopy (*n* = 3). These data were obtained from three independent experiments, and the values represent the means ± SEM. One-Way ANOVA *t*-test. *p < 0.05, **p < 0.01 and ***p < 0.001. **D** Heatmap showing the expression profiles of DNA damage response signatures derived from RNA sequencing data of CT26^shNC^ and CT26^shARID1A^ tumors treated with RT and CHK1 inhibitor. **E** Heatmap showing the immune-related gene expression profiles derived from RNA sequencing data of CT26^shNC^ and CT26^shARID1A^ tumors treated with RT and CHK1 inhibitor. **F** The resected tumors were homogenized, and the expression level of PD-L1 was analyzed by western blot (*n* = 3). **G** Tumor growth of CT26^shNC^ and CT26^shARID1A^-driven colon carcinoma established in BALC/c mice (*n* = 3/group) that were treated with RT (5 Gy for 1 fraction), CHK1 inhibition (CCT244747, 10 mg/kg) and anti-PD1 antibodies (5 mg/kg) on the indicated day. Tumor growth is reported as the mean tumor volume ± SD. *p < 0.05 and **p < 0.01. CR: complete response. Two-way ANOVA *t*-test. **H** The survival period was recorded until the endpoint was achieved. Tumor diameter >2.0 cm, tumor volume >2000 mm^3^ and death were defined as endpoint. **I** The representative images of tumor ARID1A, p-CHK1^S345^, ssDNA, and tumor PD-L1 in colorectal cancer patients. **J** The ssDNA (left) and PD-L1 (right) expression in ARID1A-High and ARID1A-Low CRC patients (*n* = 279). **K** The schematic diagram of this study.

We comprehensively evaluated the immune cell profiles within the tumor microenvironment by determining the proportions of dendritic cells (CD86^Hi^CD11c^+^ DCs and CD80^Hi^CD11c^+^ DCs), T cells (CD4^+^ T cells and CD8^+^ T cells), effector/memory T cells (CD44^+^CD62L^−^CD8^+^ T_EM_ cells), cytotoxic T cells (IFNγ^+^CD8^+^ T cells and TNFα^+^CD8^+^ T cells) and regulatory T cells (CD127^+^CD25^+^CD4^+^ Treg cells). The gating strategies are shown in Fig. S5. We found that combined radiotherapy and CHK1 inhibition can promote a more favorable immune environment by increasing the presence of antigen-presenting dendritic cells and cytotoxic T cells, which are key players in antitumor immunity in ARID1A-deficient CRC tumors. The elevation of CD86^+^ and CD80^+^ DCs indicated enhanced antigen presentation capabilities, potentially leading to more robust T-cell activation (Fig. 5D and E). Indeed, the density of CD4^+^ and CD8^+^ tumor-infiltrating lymphocytes (TILs) was also remarkably increased in the ARID1A-deficient CRC tumors that received RT and CHK1 inhibitor treatment (Fig. 5F). Moreover, the significant increase in effector/memory and cytotoxic T-cell populations, including those producing IFNγ and TNFα, also highlights a shift towards a more active immune response capable of targeting and destroying tumor cells (Fig. 5G–I). But we did not find a significant change in regulatory T cells (Fig. 5J), suggesting that the immune-stimulating effects of this combination therapy are not counterbalanced by a rise in immunosuppressive cell populations, which could otherwise dampen the antitumor response. We further examined CD3^+^ and CD8^+^ T cells in tumor sections using immunofluorescence staining. As shown in Fig. 6A–C, the density of CD3^+^ and CD8^+^ T cells was greater in the CT26^shARID1A^/RT/CHKi subgroup (Fig. 6B and C), compared to the CT26^shNC^/RT/CHKi subgroup. These results indicated that blockade of CHK1 in combination with RT significantly remodeled the tumor microenvironment to recruit immune cells and induce antitumor immunity in an ARID1A-deficient CRC animal model. Furthermore, transcriptomic analysis of resected tumors also revealed that, in ARID1A-deficient cells, combined treatment with RT and CHK1 inhibition significantly increased genes associated with the DNA damage response (DDR) pathway (Fig. 6D). These results indicated that DNA damage induced by RT and CHK1 inhibition can lead to the accumulation of cytosolic DNA fragments. These fragments activated the cGAS-STING pathway to trigger the production of type I IFNs, which created a proinflammatory milieu that promotes the infiltration of immune cells, particularly dendritic cells and cytotoxic T lymphocytes, in ARID1A-deficient CRC tumors.

### CHK1 inhibition increases the susceptibility to ICBs in combination with local radiotherapy in an ARID1A-deficient CRC animal model

The results from RNA sequencing revealed that greater immune cell infiltration in the CT26^shARID1A^/RT/CHKi subgroup compared to the CT26^shNC^/RT/CHKi subgroup (Fig. 6E). These findings suggested that radiotherapy and CHK1 inhibition may enhance the recruitment of immune cells into the tumor microenvironment. However, we found that increased PD-L1 expression in residual tumor tissues, which may attenuate T-cell activity and limit antitumor immune response (Fig. 6F). Therefore, we employed a triple combination treatment approach with radiotherapy, CHK1 inhibitor, and anti-PD1 antibodies to block the PD1/PD-L1 cascade in CT26^shARID1A^ tumors (Fig. 6G). We found that this therapeutic regimen significantly delayed tumor growth, led to complete tumor regression and prolonged overall survival (Fig. 6G and H). These results indicated that triple treatment of CHK1 inhibitor, ICB and RT can meaningfully benefit ARID1A-deficient CRC patients. To demonstrate the clinical relevance of these correlations, we evaluated the expression level of ARID1A, p-CHK1^S345^, ssDNA and tumor PD-L1 in CRC patients by immunohistochemical analysis (Fig. 6I). Consistent with previous results, the level of ARID1A was negatively correlated with p-CHK1. Moreover, we also found that the level of ARID1A was negatively correlated with ssDNA and tumor PD-L1 level (Fig. 6I and J). Taken together, these results showed that targeting CHK1 may reinvigorate the RT-induced T cell response within the TME by ssDNA-mediated cGAS/STING activation for type I IFN production in ARID1A-deficient CRC patients.

## Discussion

ARID1A is the most frequently mutated subunit of the SWI/SNF chromatin remodeling complex in cancer [50]. In this study, we detected low expression of ARID1A (~40%) in primary CRC tumors. Moreover, the loss of ARID1A impaired CHK1-mediated DNA repair by DDB1-dependent proteasomal degradation, leading to heightened radiosensitivity and the accumulation of cytosolic ssDNA. Targeting CHK1 significantly increased the sensitivity to radiotherapy and the accumulation of cytosolic ssDNA, promoting cGAS/STING-mediated type I IFN production via TBK1/IRF3 and increasing cancer immunogenicity to recruit dendritic cells and enhance their maturation for the T-cell response in ARID1A-deficient tumors (Fig. 6K). Additionally, CHK1 inhibition in combination with RT markedly increased the therapeutic response of ARID1A-deficient tumors to ICBs. Taken together, these results suggested that targeting CHK1 may be a potential therapeutic strategy for ARID1A-deficient tumors in combination with RT and ICBs.

Several studies have reported that ARID1A expression is lost in CRC, but the mechanisms that lead to ARID1A silencing are still unknown. Here, we found that heterologously expressed ARID1A was present in primary CRC tumors (~40% low ARID1A expression) and was significantly downregulated in CRC tumors compared with adjacent normal tissues. Although ARID1A mutations are a primary cause of ARID1A protein loss in ~10–15% of CRC patients, other mechanisms- such as epigenetic regulation and microRNAs-mediated repression- also contribute to ARID1A downregulation [8, 51]. Recent elegant studies by Erfani et al. revealed that DNA methylation of the ARID1A promoter limits its expression in CRC patients [51, 52]. They reported that ~60% of CRC patients express no or low levels of the ARID1A protein, which was consistent with our studies. Furthermore, we found that low ARID1A expression was related to clinicopathological parameters and the survival status, which was consistent with previously published studies [8, 51]. Moreover, ARID1A deficiency is associated with diverse therapeutic responses to chemotherapy [53], radiotherapy [42], targeted therapy [54] and ICBs [16, 55]. ARID1A deficiency leads to resistance to chemotherapy and cetuximab in patients with lung cancer and CRC through multiple mechanisms [53, 54]. However, due to ARID1A deficiency contributing to impaired MMR, dysfunctional DDR and DNA checkpoint protein expression, ARID1A-deficient tumors are selectively vulnerable to radiotherapy and DNA damage inhibitors, such as PARP inhibitor [12, 42], ATR inhibitor [56] and CHK2 inhibitor [21]. These impaired MMR capacities increase cancer mutability, leading to a higher load of somatic mutation-derived neoantigens and thereby promoting immune cell infiltration and enhancing the therapeutic efficacy of ICB therapy [12, 13, 31]. Furthermore, ARID1A-deficient cancer cells also exhibit elevated type I IFN response signaling, promoting CD8^+^ T-cell recruitment and cytolytic activity [14]. Consistent with these previous studies, our results showed that ARID1A knockdown sensitized cells to CHK1 inhibitor CCT244747 and radiotherapy. Cancer cell-intrinsic type I IFN levels were also dramatically increased by radiation and CHK1 inhibition through the cGAS/STING axis, which in turn reshaped the tumor microenvironment within ARID1A-deficient tumors. By investigating ARID1A-deficient resected tumors after RT/CHKi treatment, we found that tumor PD-L1 expression was also increased, which is another hallmark of the ICB response. In support of our findings, ARID1A deficiency promoted PD-L1 upregulation by PI3K/AKT signaling to enhance the response to ICBs [15, 16, 55]. Therefore, the combination of radiotherapy and CHK1 inhibition might be a therapeutic strategy for immunotherapy in ARID1A-deficient tumors.

CHK1/2 inhibition has recently been reported to potentiate the antitumor immune response and sensitize cells to ICBs, such as glioblastoma multiforme, head and neck squamous cell carcinoma, and small cell lung cancers [18, 19, 57–59]. ARID1A deficiency might result in a replication stress response by CHK1 upregulation, leading to an increased basal level of cytosolic ssDNA. Pharmacological induction of replication stress responses with a CHK1 inhibitor was shown to increase the benefits of radiotherapy and ICBs by increasing cancer immunogenicity and remodeling the TME. Since the ATR/CHK1 DNA damage response pathway becomes activated by exposure to ssDNA, we believe that the pharmacological inhibition of CHK1 leads to cytosolic ssDNA accumulation to augment cGAS/STING-mediated type I IFN production for the T-cell response [46]. Consistent with our results, Wang et al. reported that inhibition of the ATM/CHK2 DNA damage checkpoint axis leads to replication stress and the accumulation of cytosolic dsDNA, which subsequently activates the STING-mediated immune response in ARID1A-deficient tumors [21]. Interestingly, they identified a novel chromatin remodeling-independent function of ARID1A, namely, regulating the E3 ligase activity of RNF8 in the context of CHK2 degradation. The loss of ARID1A increases RNF8 autoubiquitination, leading to increased CHK2 protein stability. In agreement with their work, we found that the level of the CHK1 E3 ligase DDB1 was significantly decreased in ARID1A-deficient tumors. Furthermore, our study showed that targeting CHK1 can amplify these immune responses in ARID1A-deficient tumors in response to RT, highlighting a potential therapeutic advantage over previous approaches. By examining the interplay between ARID1A mutations, DNA damage responses, and immune activation, we expand the current understanding of the molecular mechanisms driving the observed clinical benefits. However, several limitations should be considered. First, the sample size in the study is relatively small, which may impact the generalizability of the results to a broader patient population. Second, although our data point to CHK1 inhibition as a key factor in amplifying the immune response, alternative pathways and mechanisms, including potential off-target effects or contributions from other DNA damage response proteins, cannot be excluded. ARID1A plays a crucial role in maintaining genomic stability by interacting with topoisomerase IIa (TOP2A), a critical enzyme involved in resolving intertwined DNA strands during replication. This interaction facilitates DNA decatenation and helps maintain proper chromatin organization and function [60]. Loss of ARID1A compromises genomic stability, resulting in TOP2A-mediated dsDNA breaks that can accumulate and contribute to tumor progression and genomic instability [61]. Furthermore, pharmacological inhibition of both TOP1 and TOP2 has been shown to trigger STING-dependent type I IFN signaling [36, 62]. This pathway plays a key role in enhancing cancer immunogenicity by promoting the recruitment and activation of immune cells within the tumor microenvironment. By linking ARID1A loss to these processes, the role of chromatin remodeling in genome maintenance and immune modulation is highlighted, offering potential targets for cancer therapy.

Taken together, our study reveals a novel therapeutic strategy involving the use of a CHK1 inhibitor to elicit a broad DNA damage response as an immunomodulatory agent in ARID1A-deficient CRC tumors, which overcomes the current challenges associated with ICBs in CRC patients.

## Supplementary information

Supplementary information

Raw data from western blot

## Data Availability

The data that support the findings of this study are available from the corresponding author Kevin Chih-Yang Huang upon reasonable request.
