# Supplementary material for: CHK1 inhibition increases the therapeutic response to radiotherapy via antitumor immunity in ARID1A-deficient colorectal cancer
Source: Cell Death Dis. 2025 Aug 1;16(1):584. doi: 10.1038/s41419-025-07912-6 (PMC12317038; doi:10.1038/s41419-025-07912-6)
Supplement: Supplementary file 1 — Supplementary information [file 41419_2025_7912_MOESM1_ESM.docx]

**Supporting information**

**CHK1 Inhibition Increases the Therapeutic Response to Radiotherapy via Antitumor Immunity in ARID1A-deficient Colorectal Cancer**

**Table S1. Clinicopathological parameters of colon carcinoma patients (n=279)**

| Clinicopathological parameters | Total no. | Tumor ARID1A | | *p* value |
| --- | --- | --- | --- | --- |
|  |  | High | Low |  |
|  | 279 | 166 | 113 |  |
| Gender |  |  |  | 0.28 |
| Female | 107 | 68 (41%) | 39 (34.5%) |  |
| Male | 172 | 98 (59%) | 74 (65.5%) |  |
| Age |  |  |  | 0.38 |
| <65 | 157 | 97 (58.4%) | 60 (53.1%) |  |
| ≥65 | 122 | 69 (41.6%) | 53 (46.9%) |  |
| pT stage |  |  |  | 0.50 |
| pT1-2 | 129 | 74 (44.6%) | 55 (48.7%) |  |
| pT3-4 | 150 | 92 (55.4%) | 58 (51.3%) |  |
| Tumor differentiation |  |  |  | 0.41 |
| Well to moderate | 252 | 148(89.1%) | 104(92%) |  |
| Poor | 16 | 12 (7.2%) | 4(3.5%) |  |
| Unknown | 11 | 6(3.6%) | 5(4.4%) |  |
| Lymphovascular invasion |  |  |  | 0.58 |
| Absent | 86 | 94 (56.6%) | 71 (62.8%) |  |
| Present | 25 | 67 (40.4%) | 39 (34.5%) |  |
| Unknown | 4 | 5 (3.0%) | 3 (2.7%) |  |
| Perineural invasion |  |  |  | 0.98 |
| Absent | 85 | 98 (59%) | 67 (59.3%) |  |
| Present | 26 | 63 (38%) | 43 (38.1%) |  |
| Unknown | 4 | 5 (3%) | 3 (2.6%) |  |
| MMR status |  |  |  | 0.005* |
| MMR-proficient | 103 | 51(30.7%) | 52 (46.0%) |  |
| MMR-deficient | 2 | 0 (0%) | 2 (1.8%) |  |
| Unknown | 174 | 115(69.3%) | 59 (52.2%) |  |

**
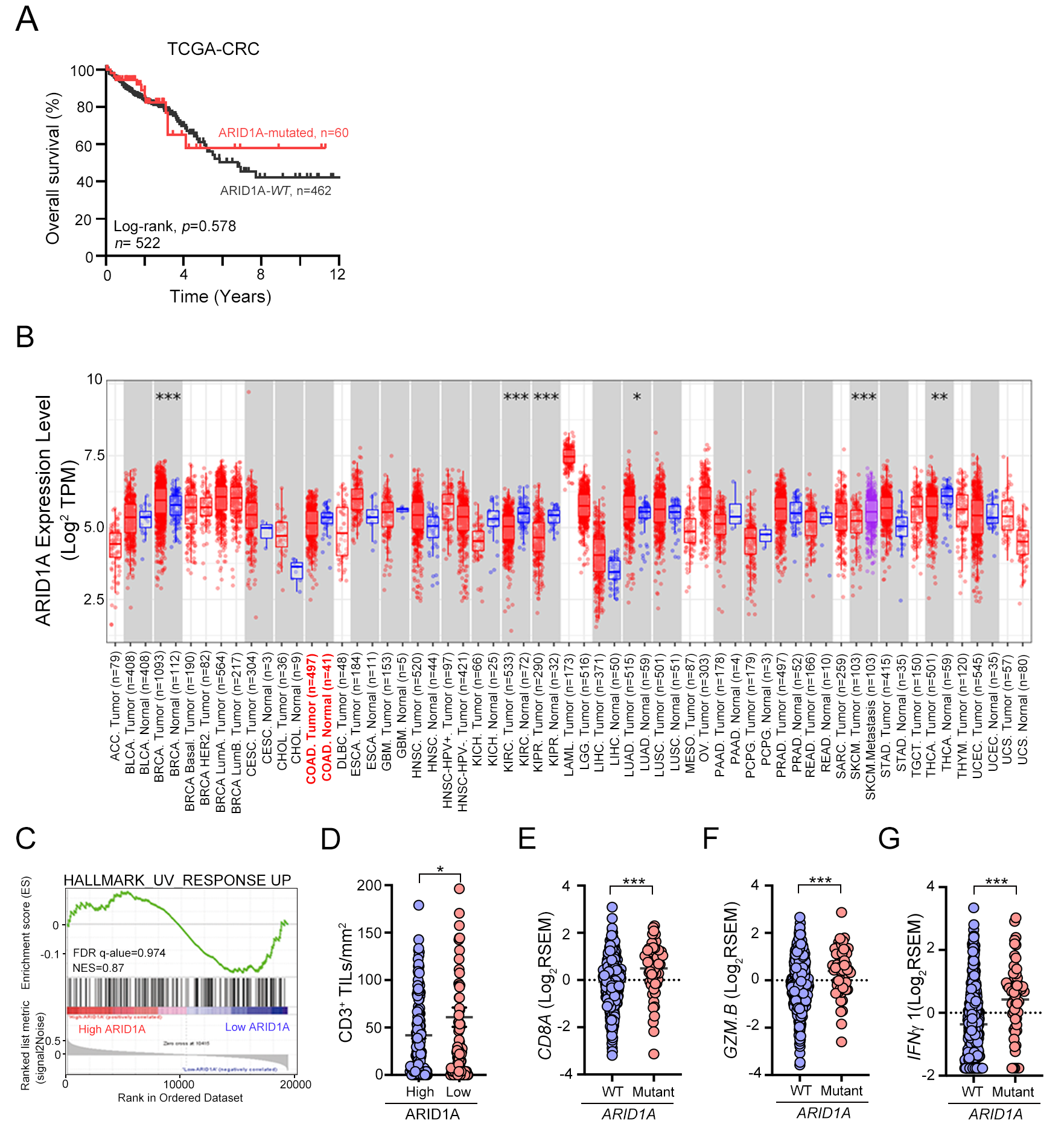
**

**Figure S1. CHK1 signaling was enhanced in CRC tumors with mutant or low ARID1A.**

1. There was no significant difference in overall survival between ARID1A-WT and ARID1A-mutated in TCGA-CRC dataset (*n*=522, Log-rank p=0.578).
2. ARID1A expression in normal versus tumor colorectal tissues, as analyzed using TIMER2.0.
3. The GSEA analysis on the HALLMARK_UV_RESPONSE_UP signature.
4. The density of tumor-infiltrated CD3^+^ T cell in ARID1A High and ARID1A Low CRC patient (*p* < 0.05, *n*=279).
5. The *CD8A* mRNA expression in ARID1A-WT and ARID1A-Mutant CRC patient (*p* < 0.001, *n* = 532).
6. The *GZM.B* mRNA expression in ARID1A-WT and ARID1A-Mutant CRC patient (*p* < 0.001, *n* = 532).
7. The *IFNγ* 1 mRNA expression in ARID1A-WT and ARID1A-Mutant CRC patient (*p* < 0.001, *n* = 532).

**
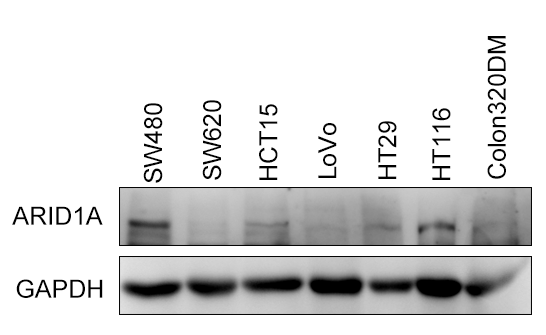
**

**Figure S2. The protein expression level of ARID1A in different colon cancer cell types.**

Whole-cell lysates were prepared and analyzed by western blotting.

**
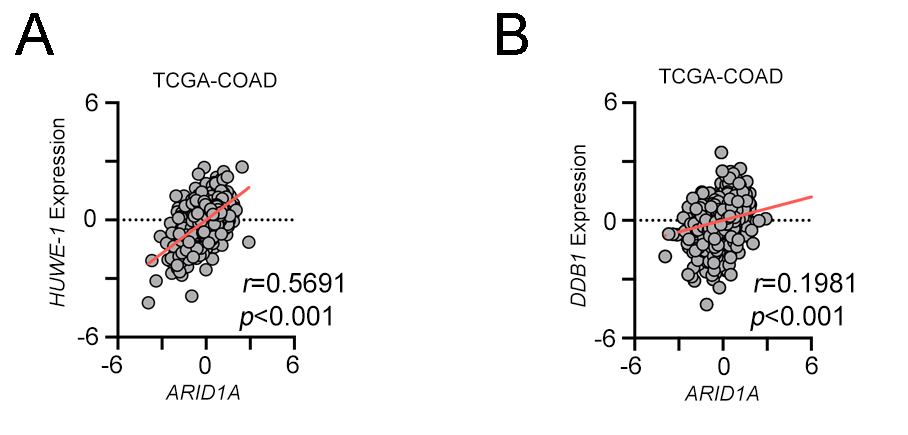
**
**Figure S3. The correlation between *ARID1A* with *HUWE-1* and *DDB1*.**

1. The correlation between *HUWE-1* and *ARID1A* mRNA expression in TCGA-COAD database (*n*=592, *p*<0.001).
2. The correlation between *DDB1* and *ARID1A* mRNA expression in TCGA-COAD database (*n*=592, *p*<0.001).

**
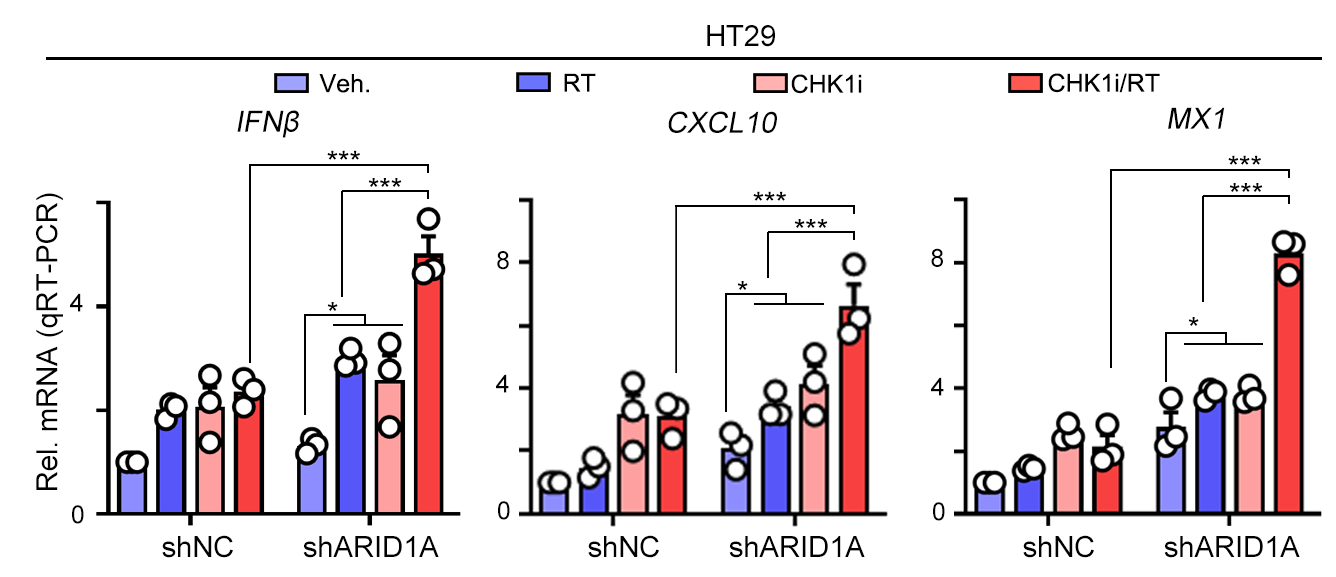
**
**Figure S4. CHK1 inhibition augmented RT-induced antitumor immunity to eradicate cancer cells in HT29 cells.**

HT29^shNC^ and HT29^shARID1A^ cells were treated with RT (5 Gy) and CHK1 inhibitor CCT244747 (10 μM) for 24 hrs. The level of type I IFN-related genes was measured by RT-qPCR (*n*=3). These data were obtained from three independent experiments, and the values represent the means ±SEM. **p*<0.05 and ****p*<0.001. One-Way ANOVA t-test.

**
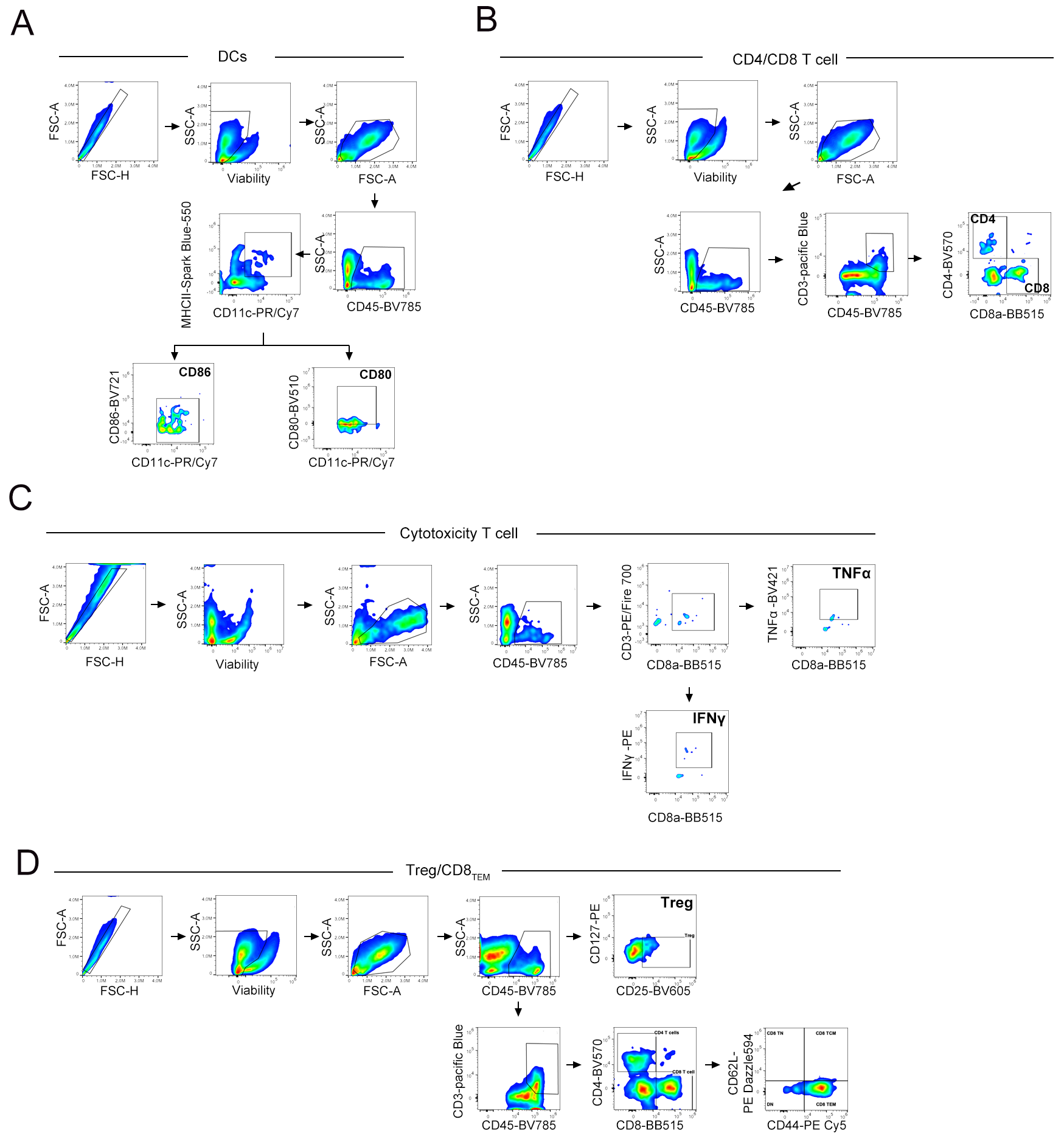

Figure S5. The gating strategy of DC and T cell subsets.**

1. The gating strategy for CD80^+^CD11c^+^ and CD86^+^CD11c^+^ dendritic cells, which is related to Fig. 5D and 5E.
2. The gating strategy for CD4^+^ and CD8^+^ CD45^+^CD3^+^ T cells, which is related to Fig. 5F.
3. The gating strategy for IFNγ^+^CD8^+^ and TNFα^+^CD8^+^ T cells, which is related to Fig. 5H and 5I.
4. The gating strategy for CD62L^+^CD44^-^CD8^+^ T effector/memory cells, which is related to Fig. 5G; the gating strategy for CD127^+^CD25^+^CD4^+^ T regulatory cells, which is related to Fig. 5J.
