# Supplementary material for: CHK1 inhibition increases the therapeutic response to radiotherapy via antitumor immunity in ARID1A-deficient colorectal cancer
Source: Cell Death Dis. 2025 Aug 1;16(1):584. doi: 10.1038/s41419-025-07912-6 (PMC12317038; doi:10.1038/s41419-025-07912-6)

CHK1 Inhibition Increases the Therapeutic Response to Radiotherapy via Antitumor Immunity in ARID1A-deficient Colorectal Cancer

Western blot RAW data

Figure 1I

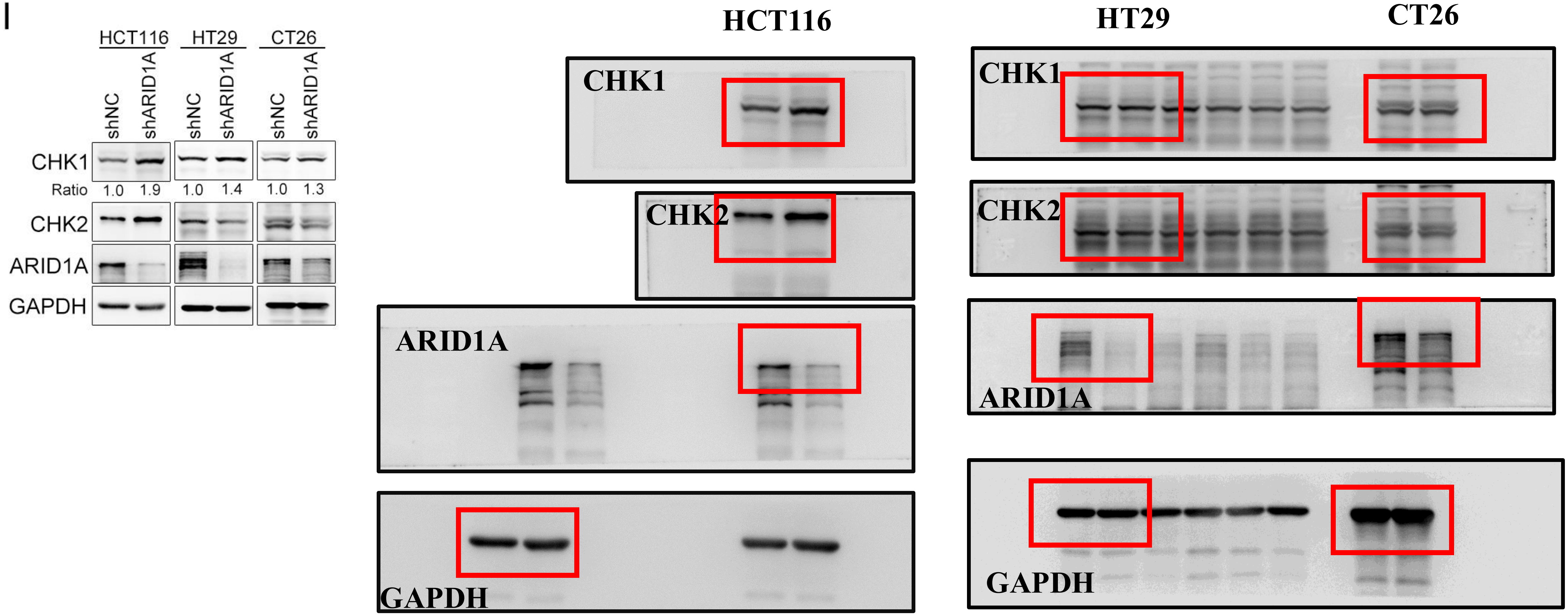

I
HCT116
HT29
CT26
shNC
shARID1A
shNC
shARID1A
shNC
shARID1A
CHK1
Ratio
1.0
1.9
1.0
1.4
1.0
1.3
CHK2
ARID1A
GAPDH
HCT116
CHK1
CHK2
ARID1A
GAPDH
HT29
CT26
CHK1
CHK2
ARID1A
GAPDH

Figure 2A

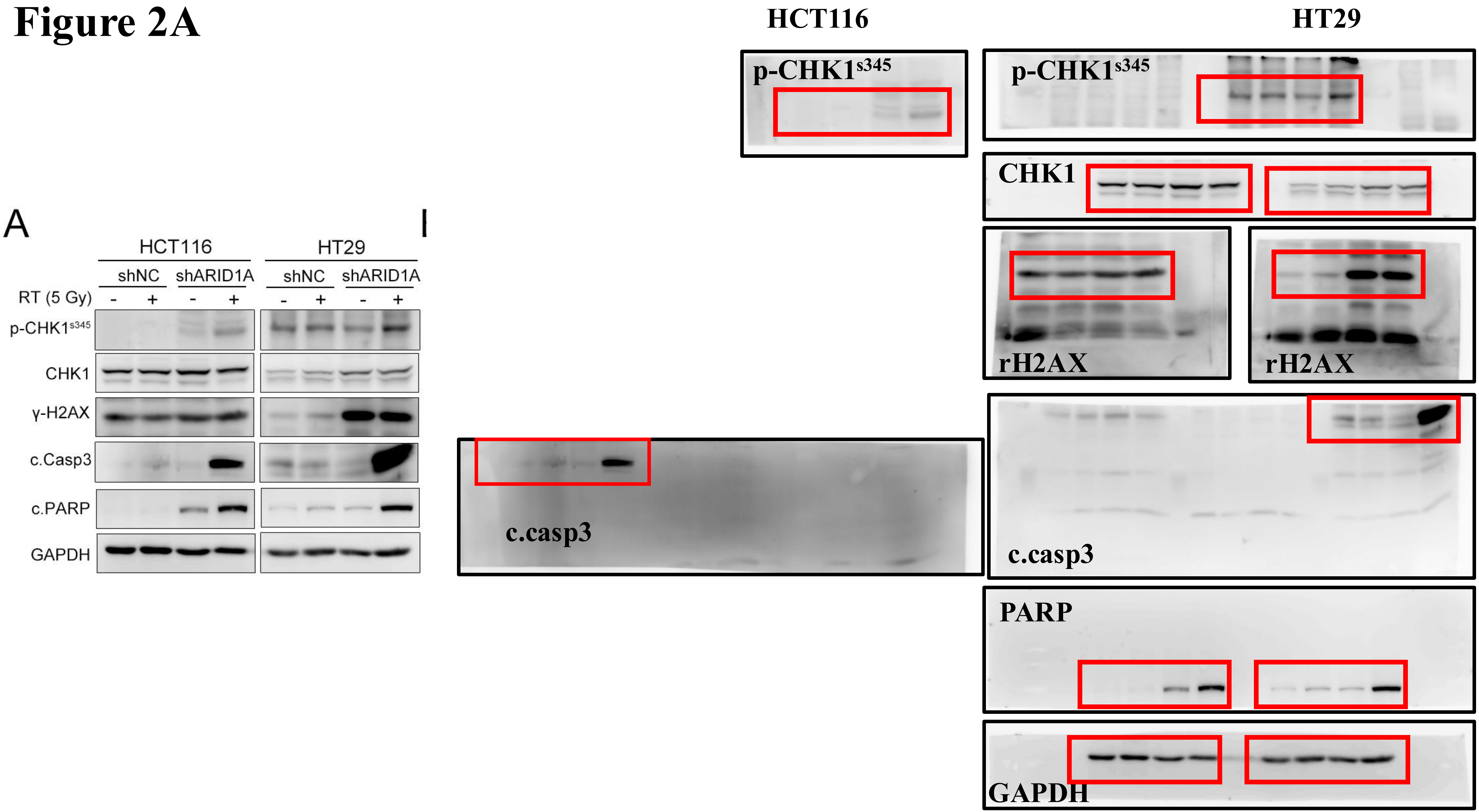
HCT116
HT29
p-CHK1s345
CHK1
rH2AX
c.casp3
PARP
GAPDH
A
shNC
shARID1A
RT (5 Gy)
γ-H2AX
c.Casp3
c.PARP

Figure 2E

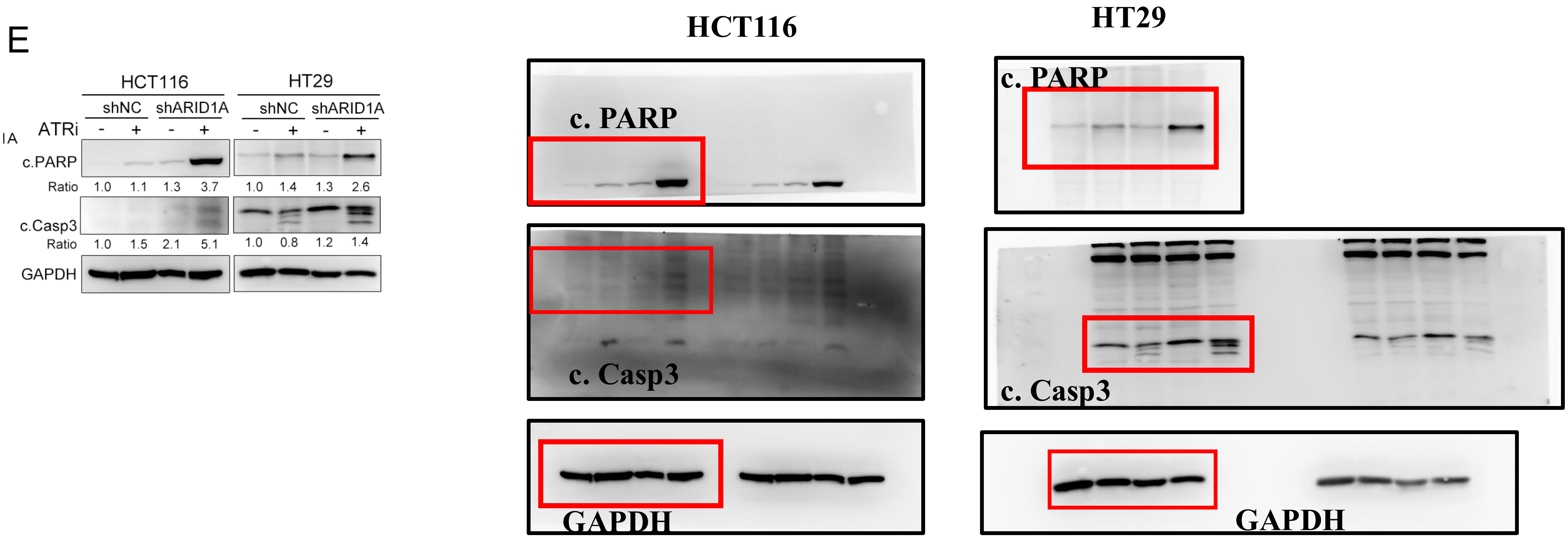
E
HCT116
HT29
shNC
shARID1A
ATRi
c.PARP
Ratio 1.0 1.1 1.3 3.7 1.0 1.4 1.3 2.6
c.Casp3
Ratio 1.0 1.5 2.1 5.1 1.0 0.8 1.2 1.4
GAPDH
HCT116
HT29
c. PARP
c. Casp3
GAPDH

Figure 2F

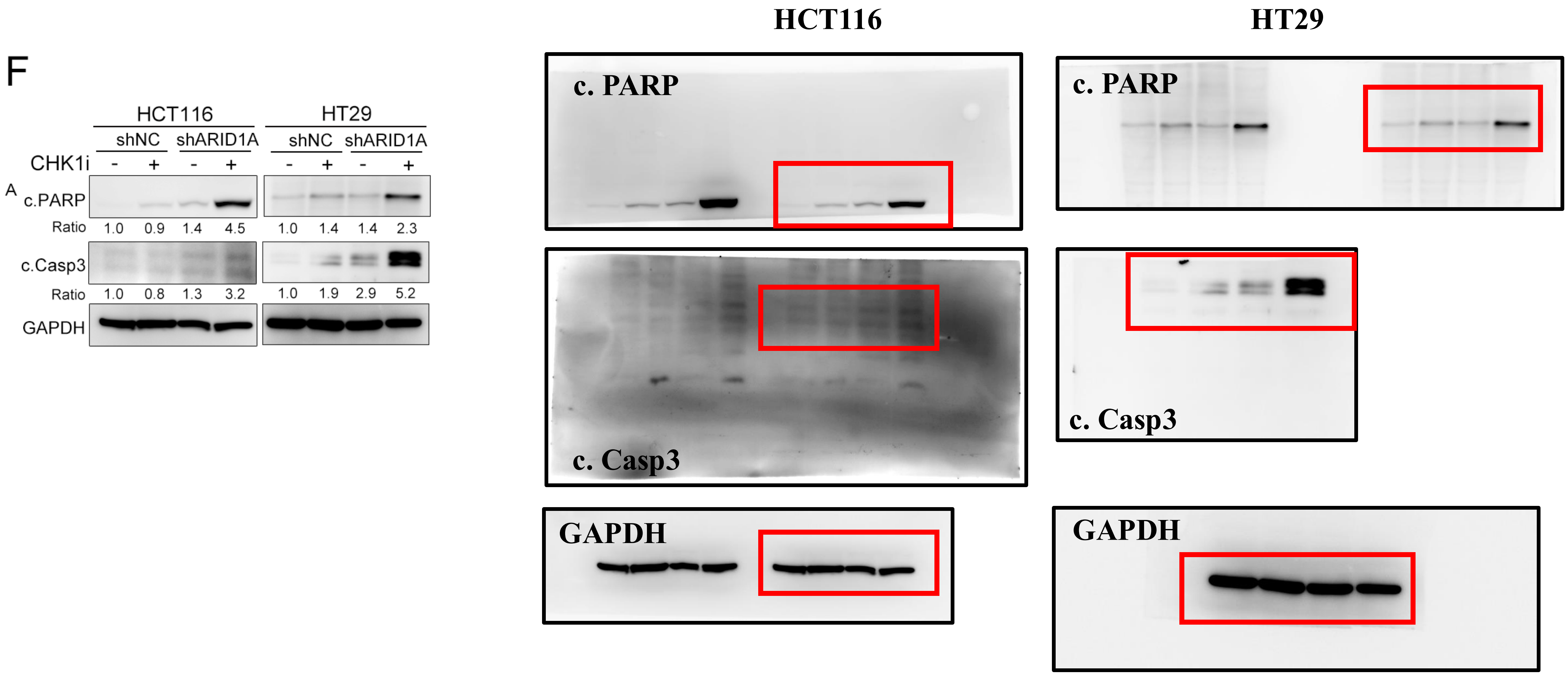
F
HCT116
HT29
shNC
shARID1A
shNC
shARID1A
CHK1i - + - + - + - +
A
c.PARP
Ratio 1.0 0.9 1.4 4.5 1.0 1.4 1.4 2.3
c.Casp3
Ratio 1.0 0.8 1.3 3.2 1.0 1.9 2.9 5.2
GAPDH
HCT116
HT29
c. PARP
c. PARP
c. Casp3
c. Casp3
GAPDH
GAPDH

Figure 2H

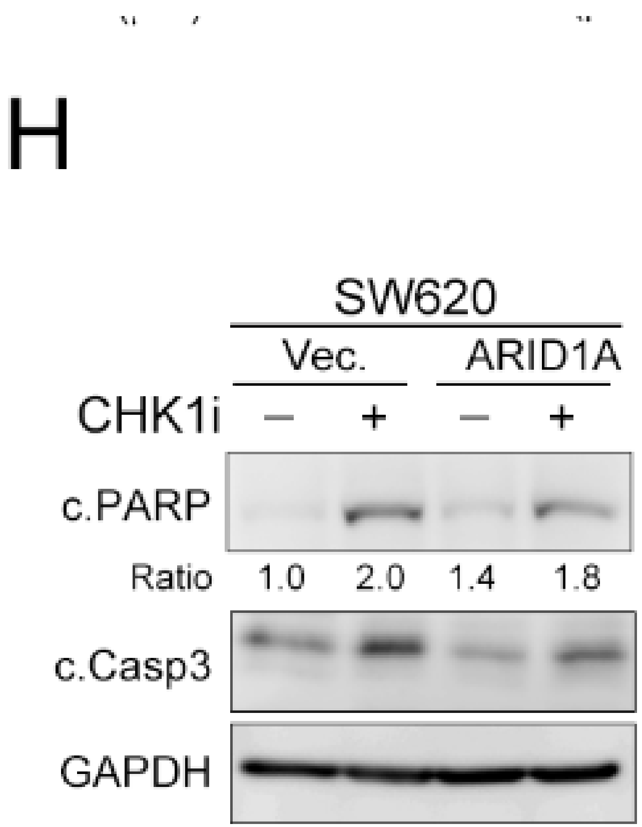
H
SW620
Vec.
ARID1A
CHK1i
–
+
–
+
c.PARP
Ratio
1.0
2.0
1.4
1.8
c.Casp3
GAPDH

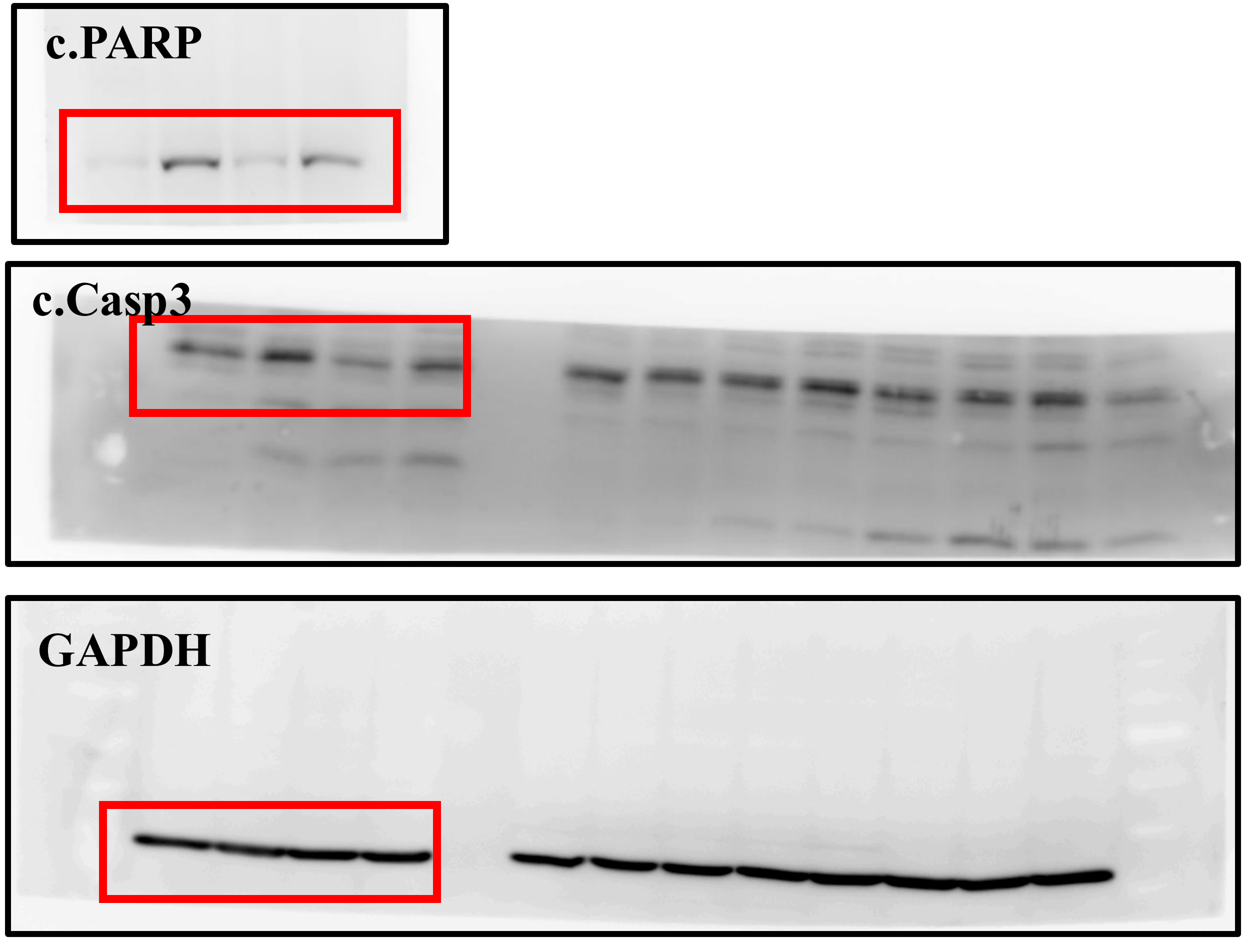
c.PARP
c.Casp3
GAPDH

Figure 2I

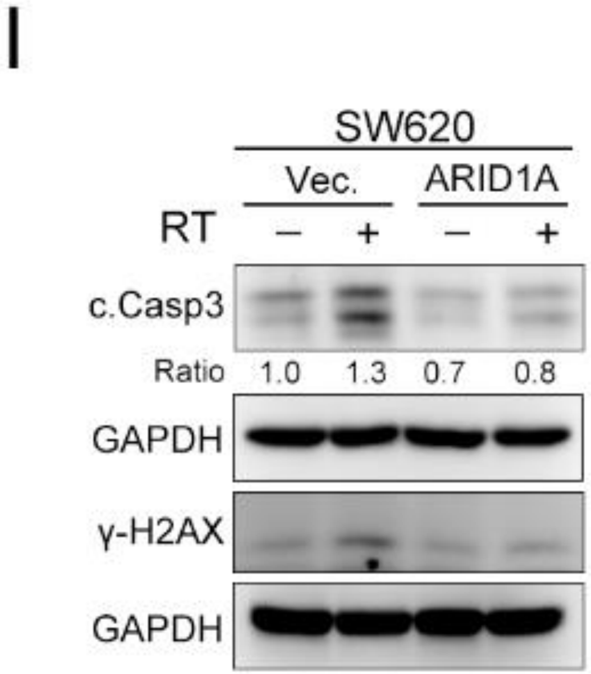

SW620

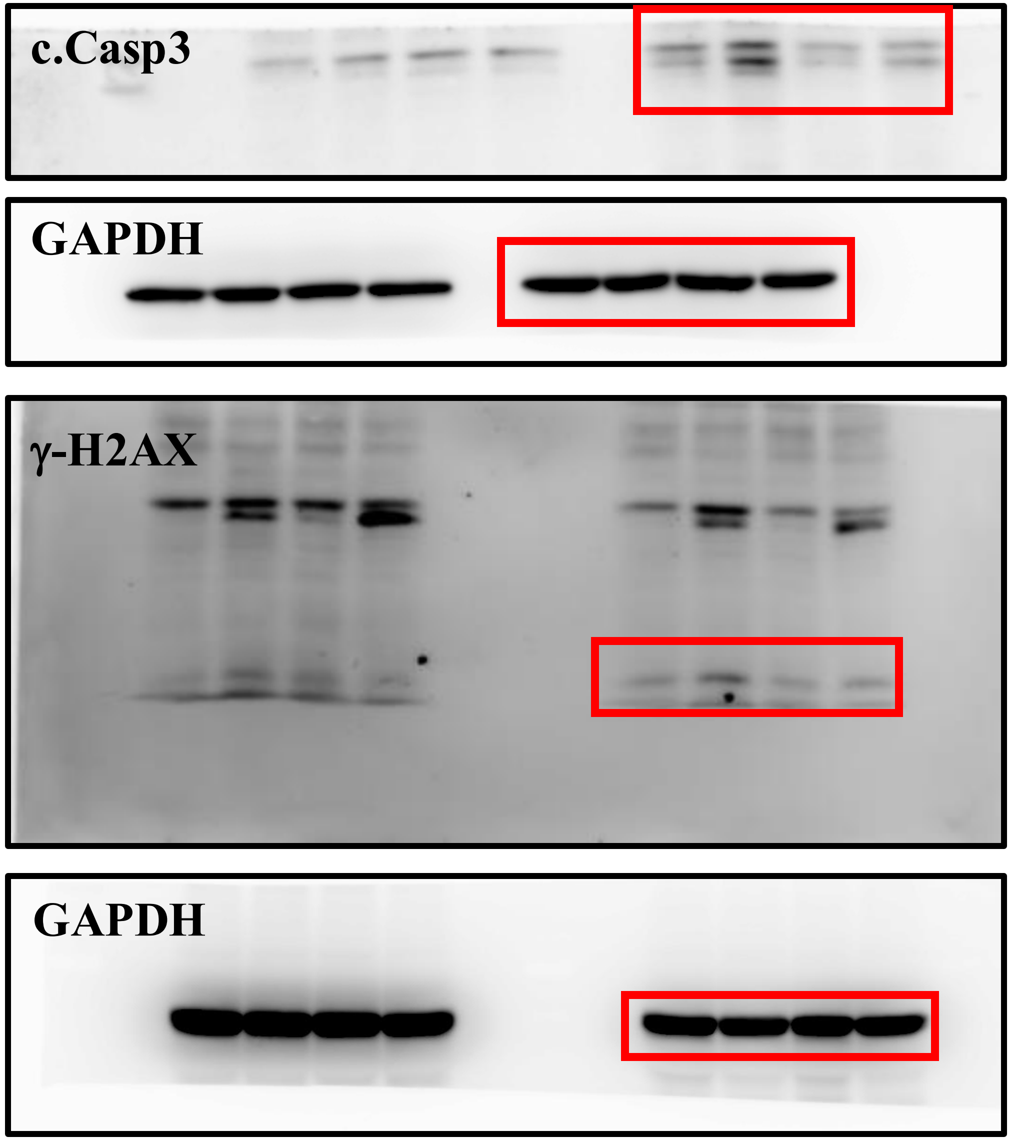

Figure 3A

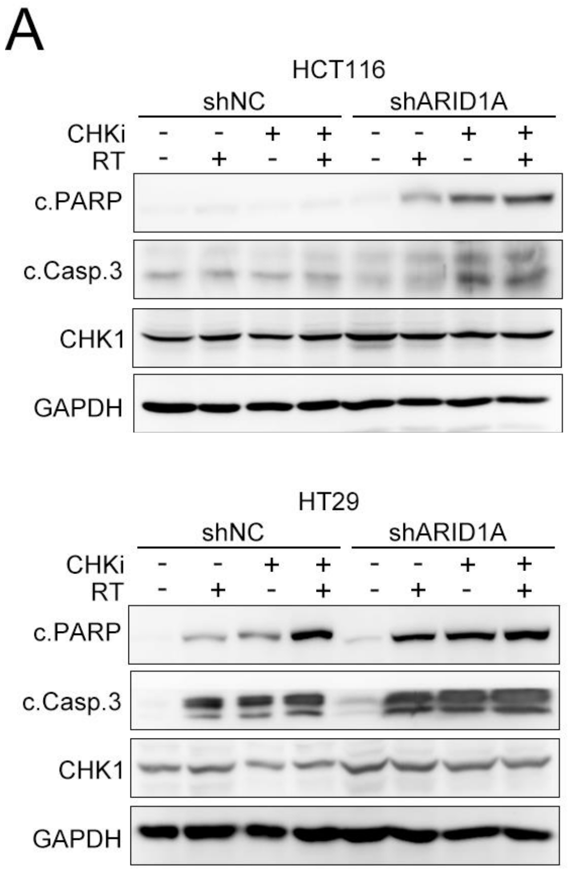
A
HCT116
shNC
shARID1A
CHKi - - + + - - + +
RT - + - + - + - +
c.PARP
c.Casp.3
CHK1
GAPDH
HT29
shNC
shARID1A
CHKi - - + + - - + +
RT - + - + - + - +
c.PARP
c.Casp.3
CHK1
GAPDH

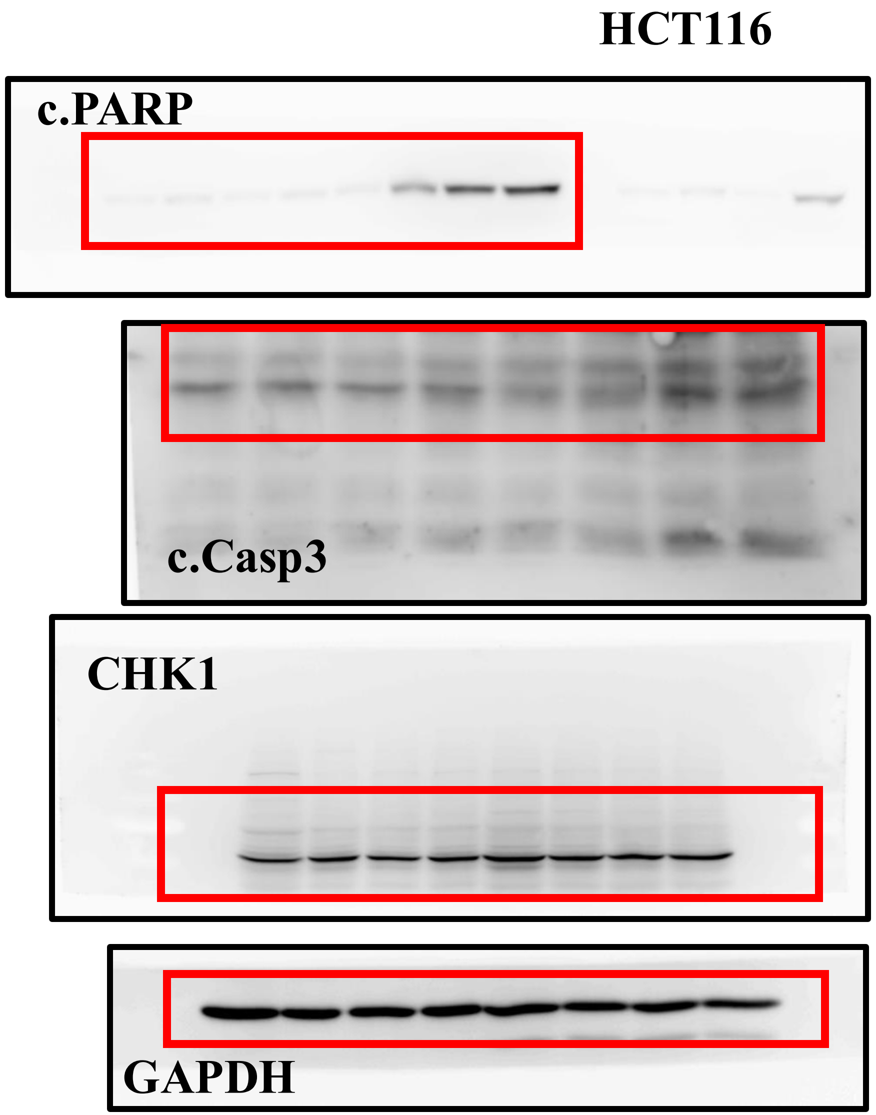
HCT116
c.PARP
c.Casp3
CHK1
GAPDH

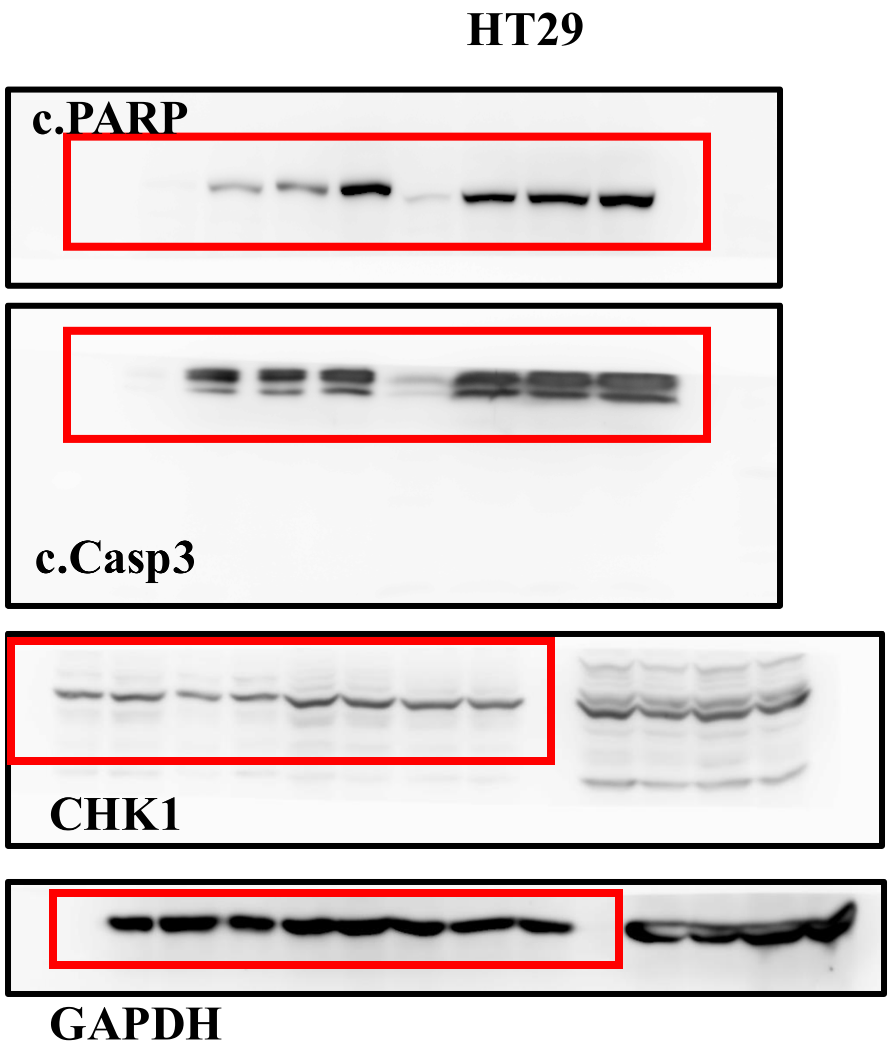
HT29
c.PARP
c.Casp3
CHK1
GAPDH

Figure 3B

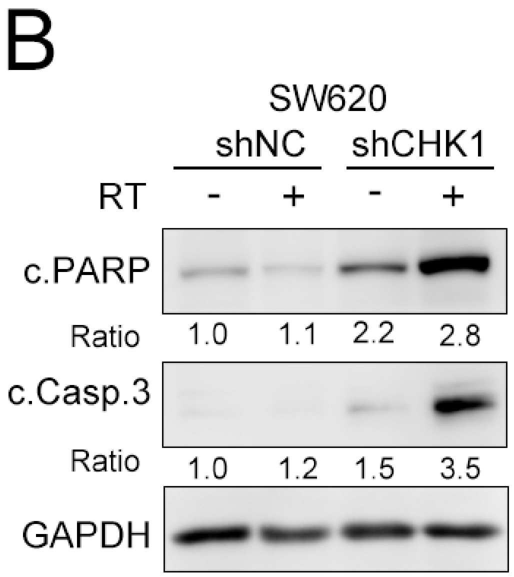
B
SW620
shNC
shCHK1
RT
-
+
-
+
c.PARP
Ratio
1.0
1.1
2.2
2.8
c.Casp.3
Ratio
1.0
1.2
1.5
3.5
GAPDH

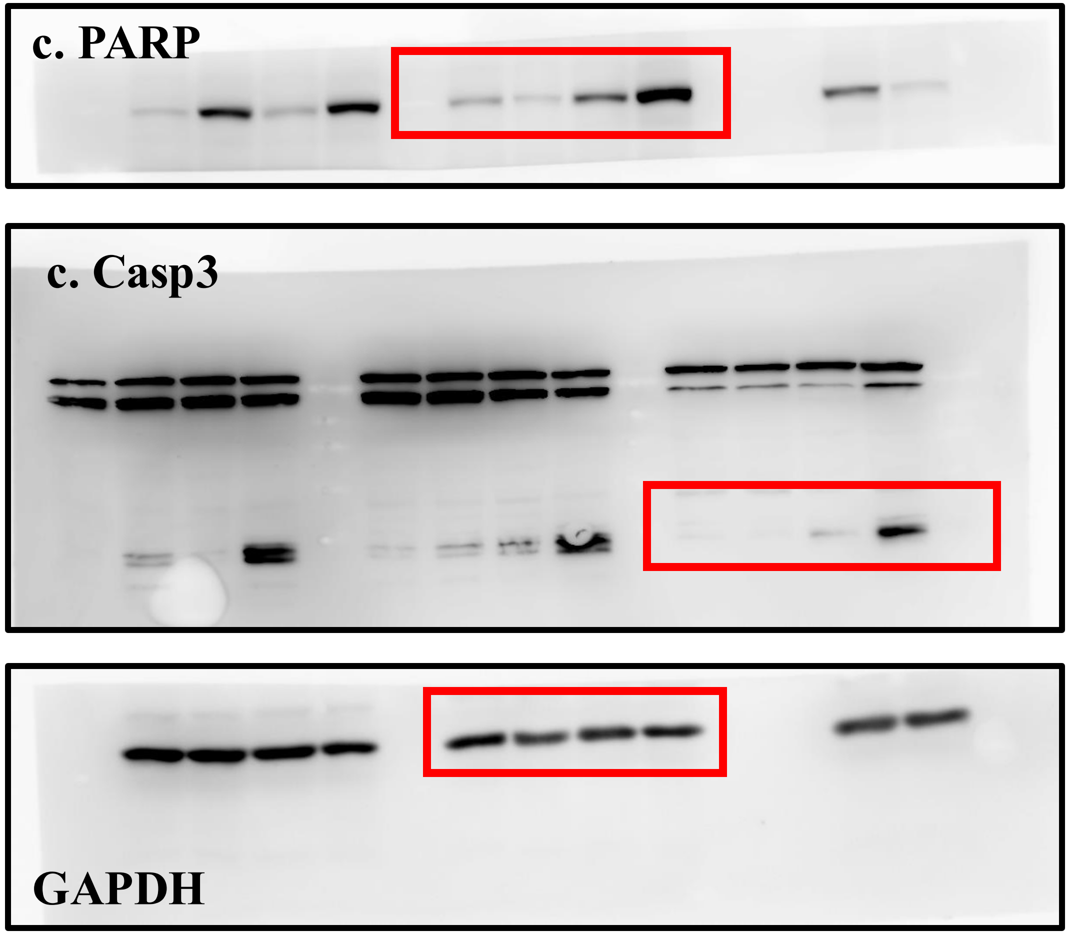
c. PARP
c. Casp3
GAPDH

Figure 3D

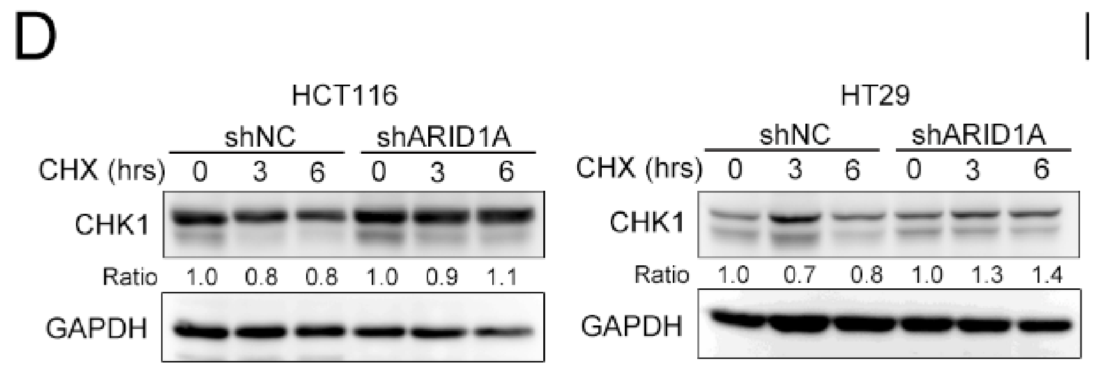
D
HCT116
shNC
shARID1A
CHX (hrs) 0 3 6 0 3 6
CHK1
Ratio 1.0 0.8 0.8 1.0 0.9 1.1
GAPDH
HT29
shNC
shARID1A
CHX (hrs) 0 3 6 0 3 6
CHK1
Ratio 1.0 0.7 0.8 1.0 1.3 1.4
GAPDH

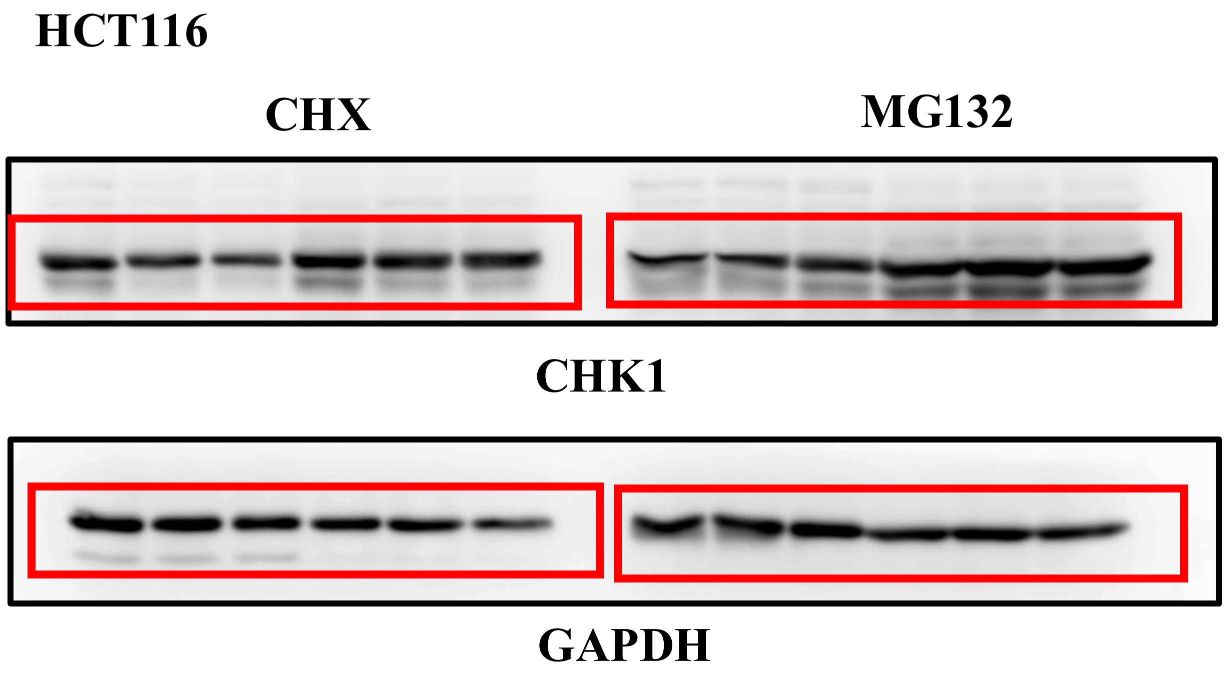
HCT116
CHX
MG132
CHK1
GAPDH

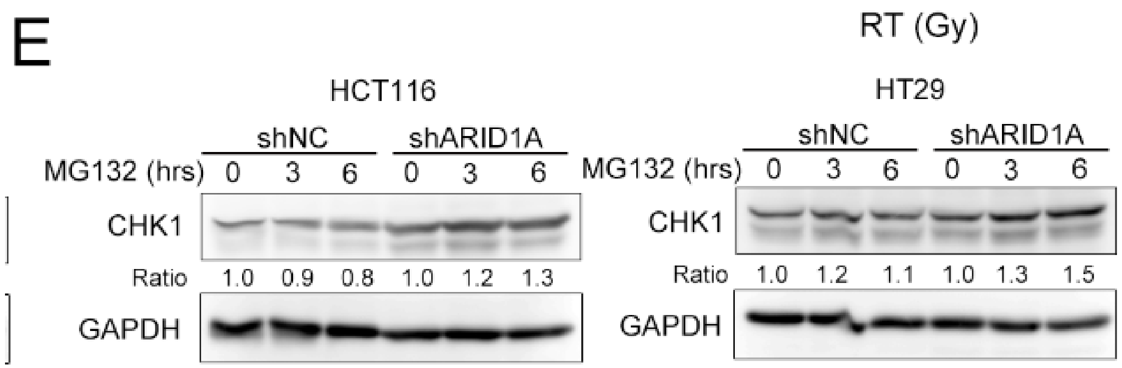
E
RT (Gy)
HCT116
shNC
shARID1A
MG132 (hrs) 0 3 6 0 3 6
CHK1
Ratio 1.0 0.9 0.8 1.0 1.2 1.3
GAPDH
HT29
shNC
shARID1A
MG132 (hrs) 0 3 6 0 3 6
CHK1
Ratio 1.0 1.2 1.1 1.0 1.3 1.5
GAPDH

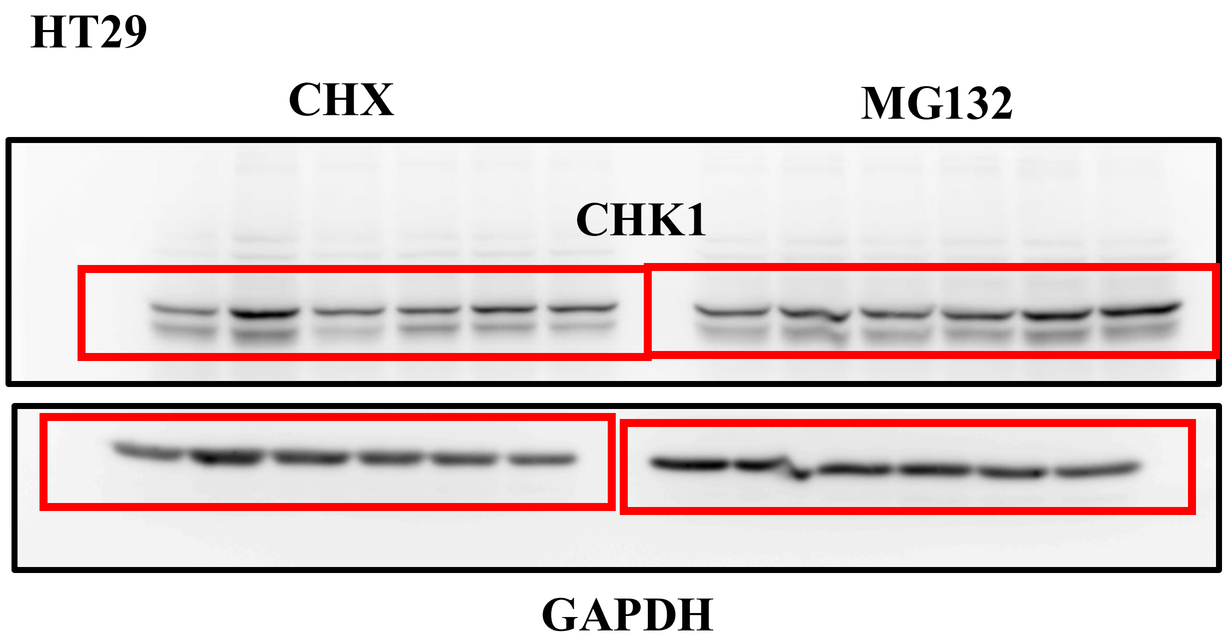
HT29
CHX
MG132
CHK1
GAPDH

Figure 3F

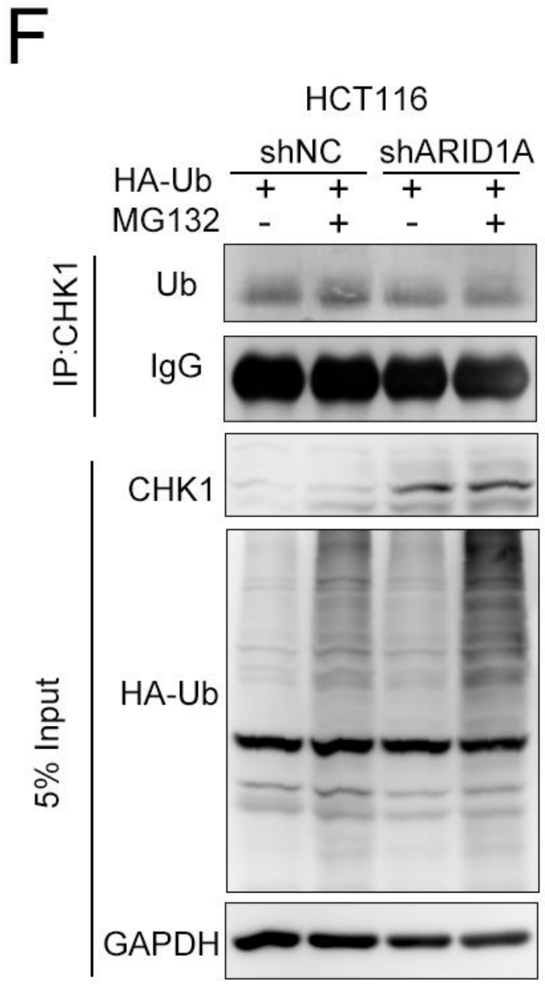
F
HCT116
shNC
shARID1A
HA-Ub
MG132
IP:CHK1
Ub
IgG
CHK1
HA-Ub
GAPDH
5% Input

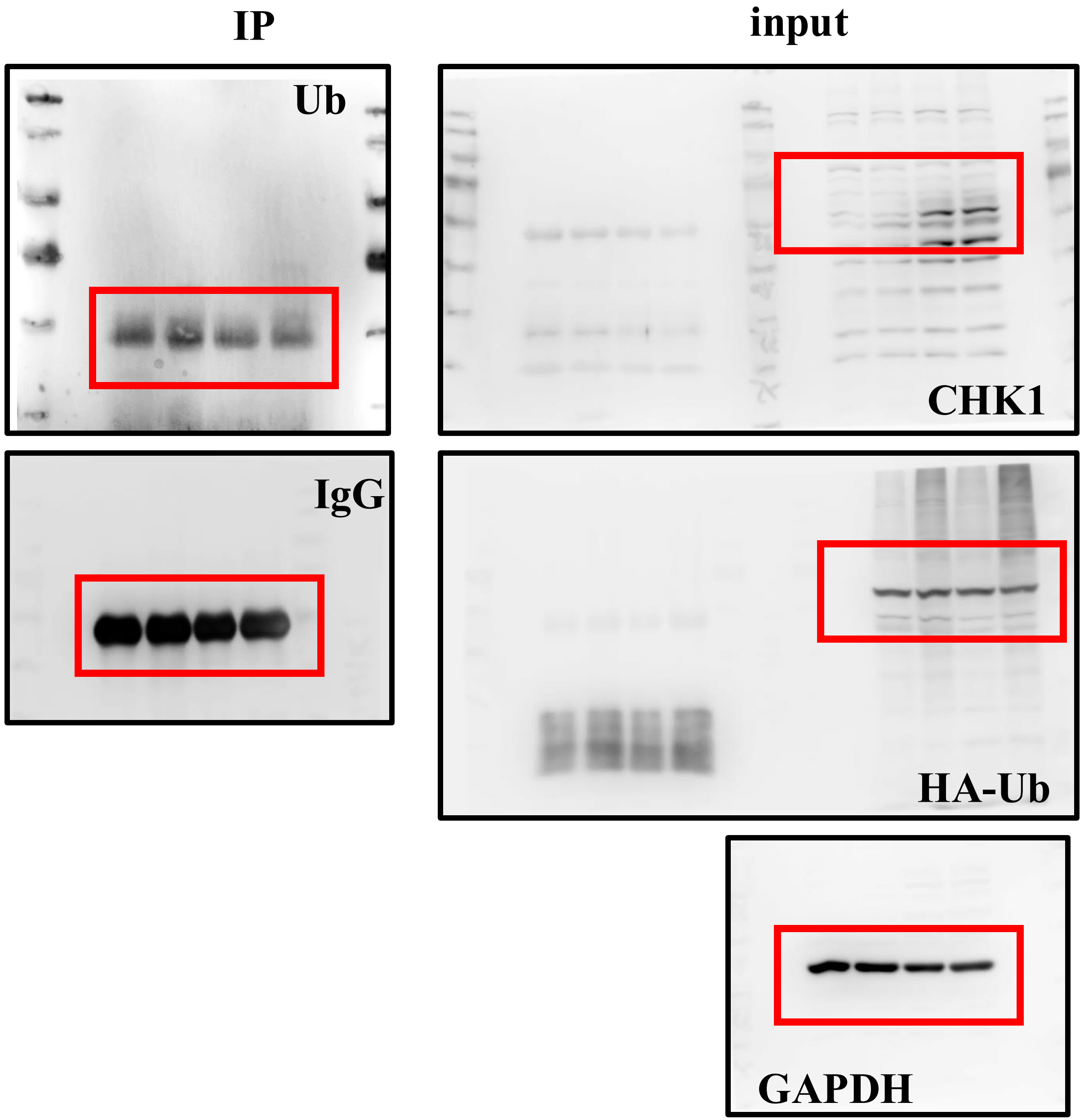
IP
input
Ub
CHK1
IgG
HA-Ub
GAPDH

Figure 3G

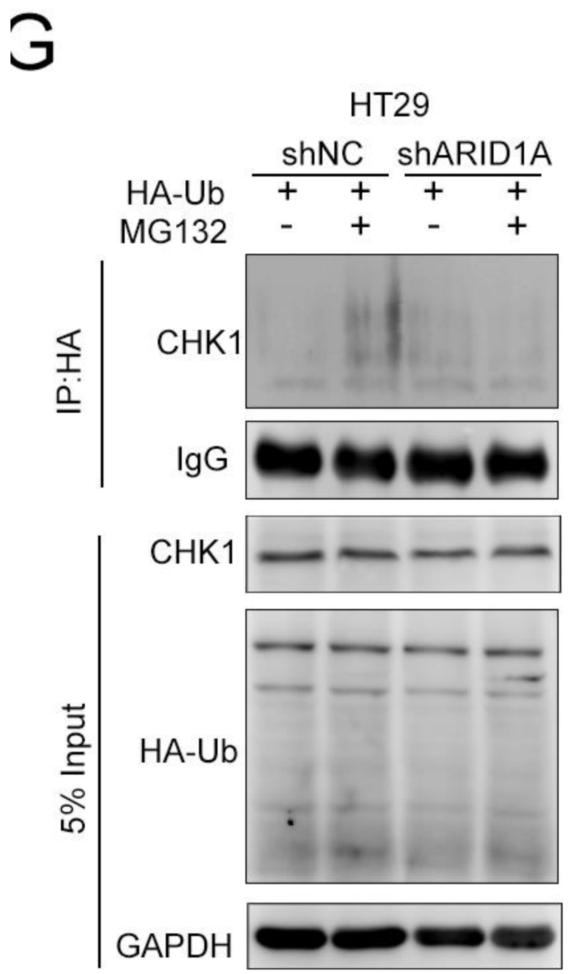
G
HT29
shNC
shARID1A
HA-Ub
MG132
IP:HA
CHK1
IgG
5% Input
CHK1
HA-Ub
GAPDH

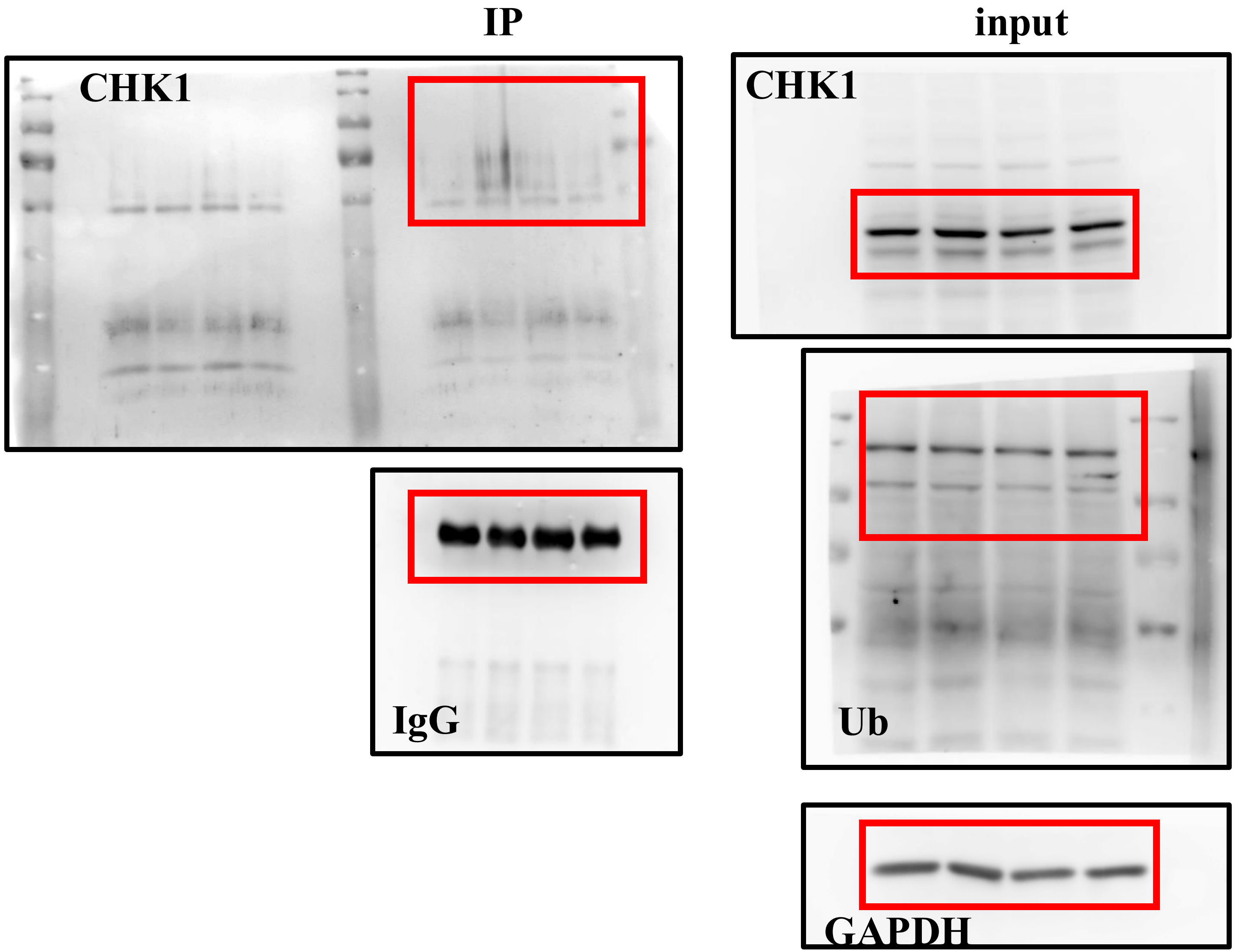
IP
input
CHK1
CHK1
IgG
Ub
GAPDH

Figure 3H

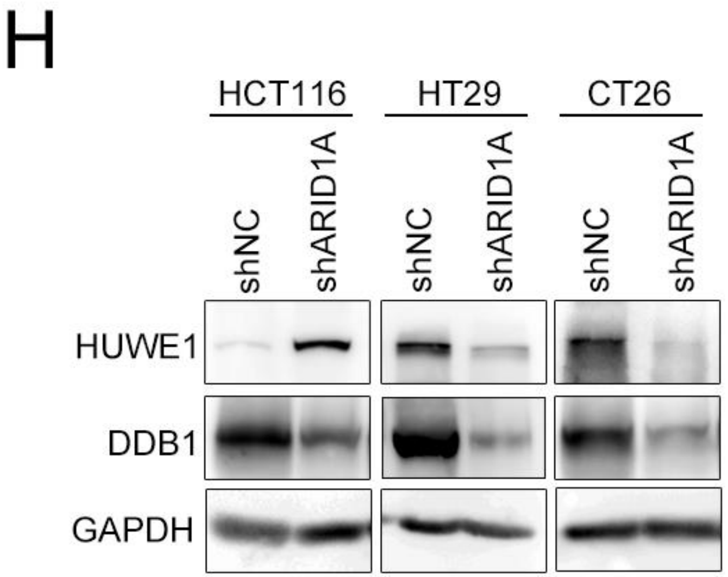
H
HCT116
HT29
CT26
shNC
shARID1A
shNC
shARID1A
shNC
shARID1A
HUWE1
DDB1
GAPDH

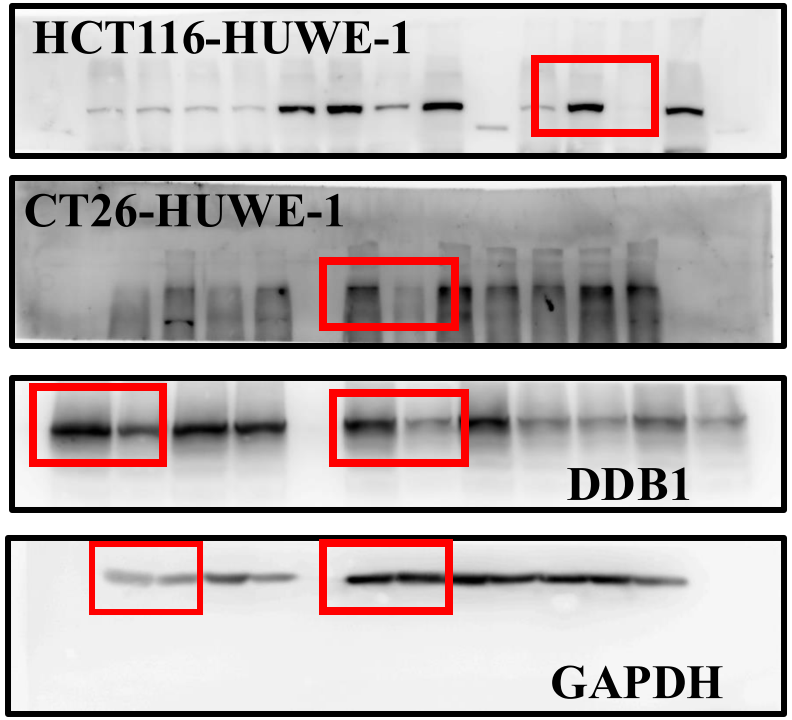
HCT116-HUWE-1
CT26-HUWE-1
DDB1
GAPDH

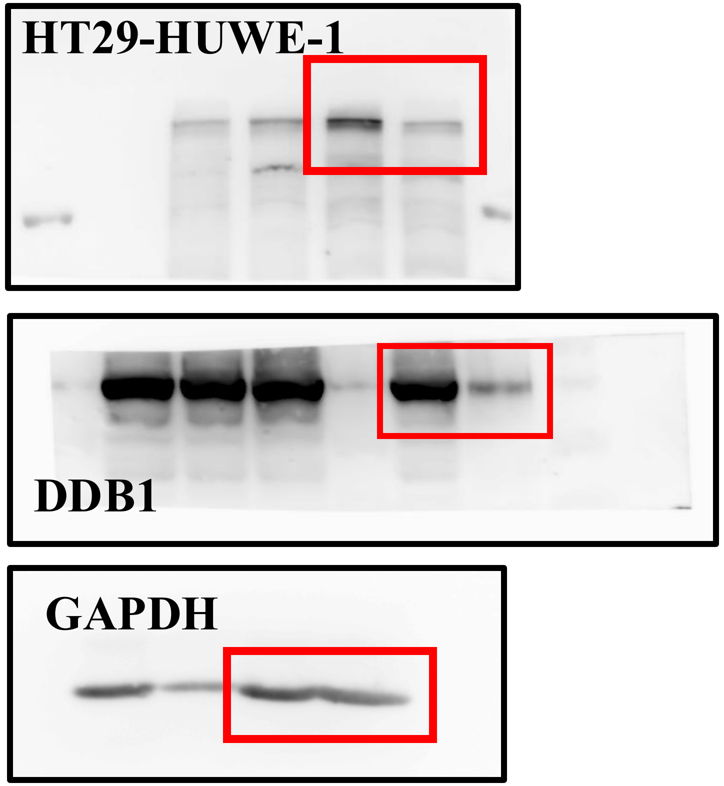
HT29-HUWE-1
DDB1
GAPDH

Figure 3I

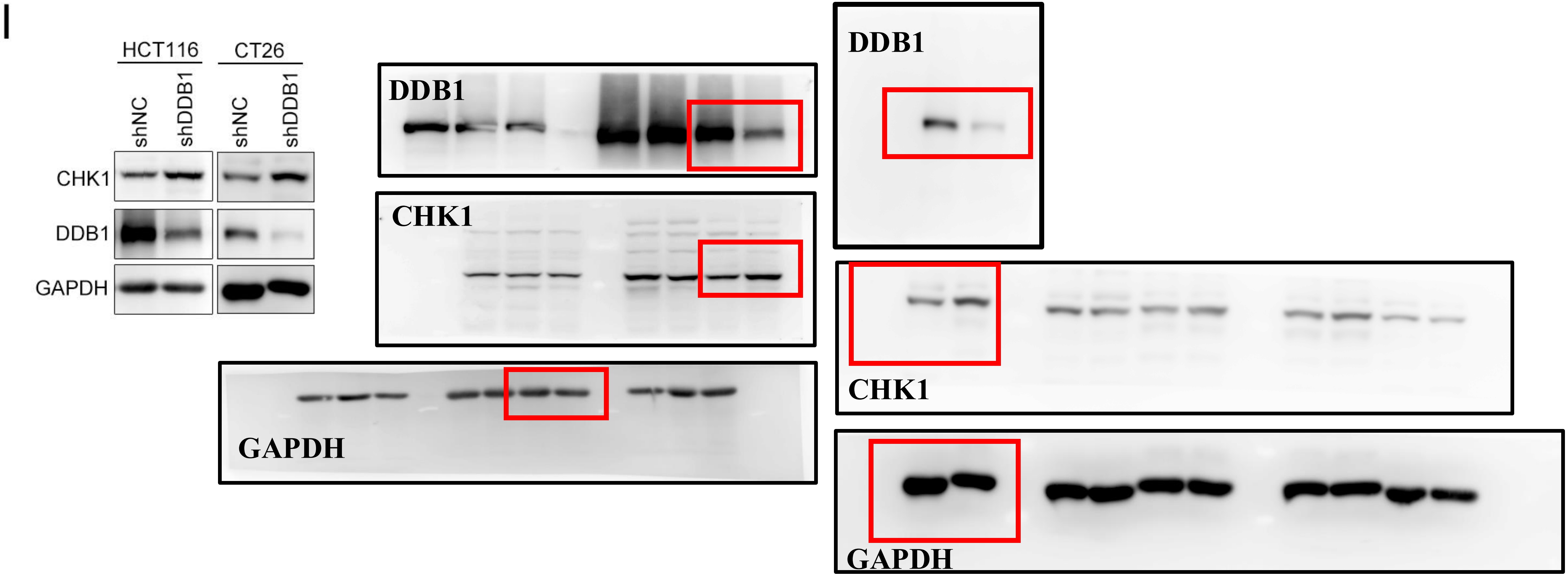
I
HCT116
CT26
shNC
shDDB1
shNC
shDDB1
CHK1
DDB1
GAPDH
DDB1
CHK1
GAPDH
DDB1
CHK1
GAPDH

Figure 3J

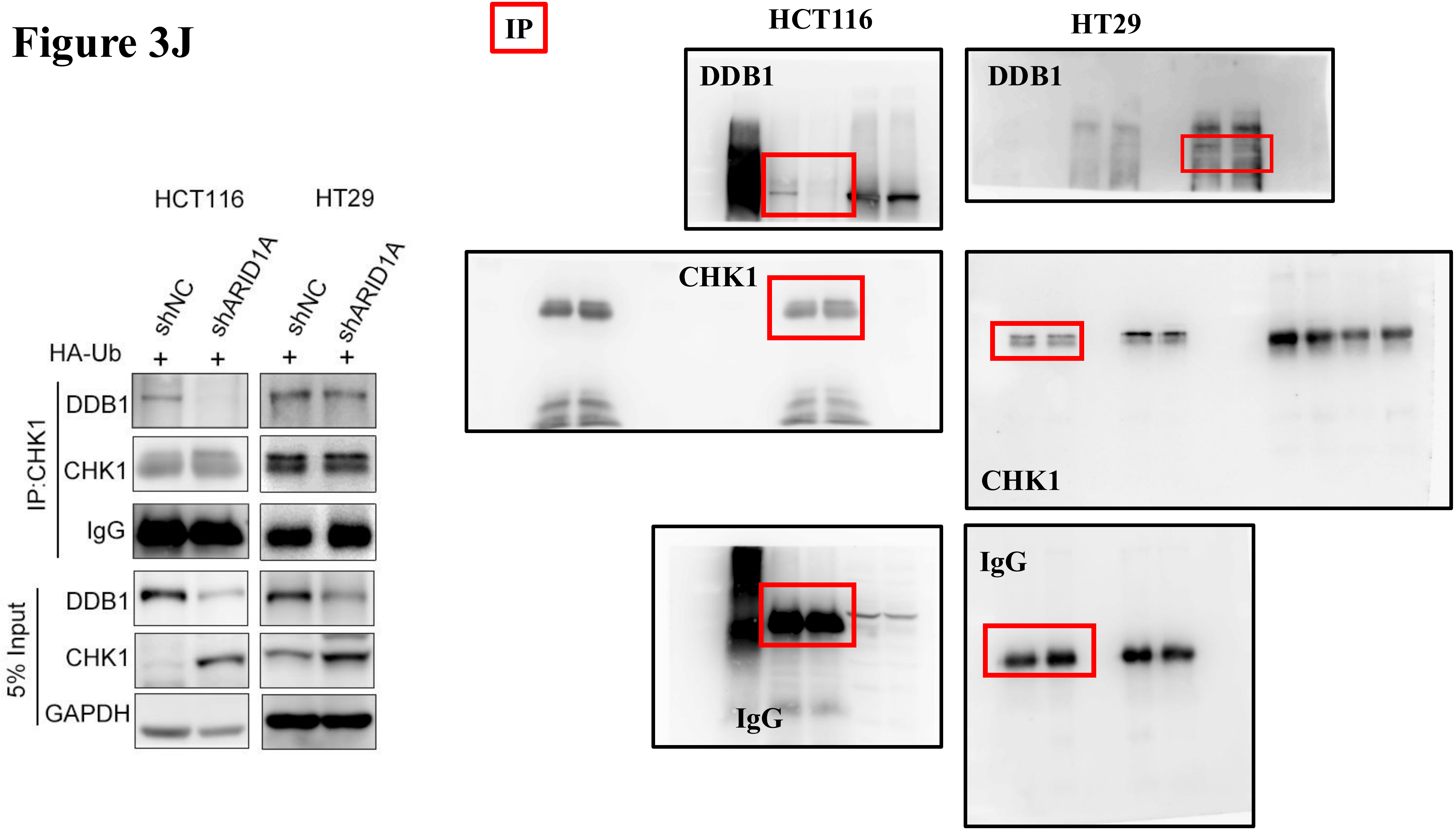

IP
HCT116
HT29
DDB1
DDB1
CHK1
CHK1
IgG
IgG
HCT116
HT29
shNC
shARID1A
shNC
shARID1A
HA-Ub
+
+
+
+
IP:CHK1
DDB1
CHK1
IgG
5% Input
DDB1
CHK1
GAPDH

Figure 3J

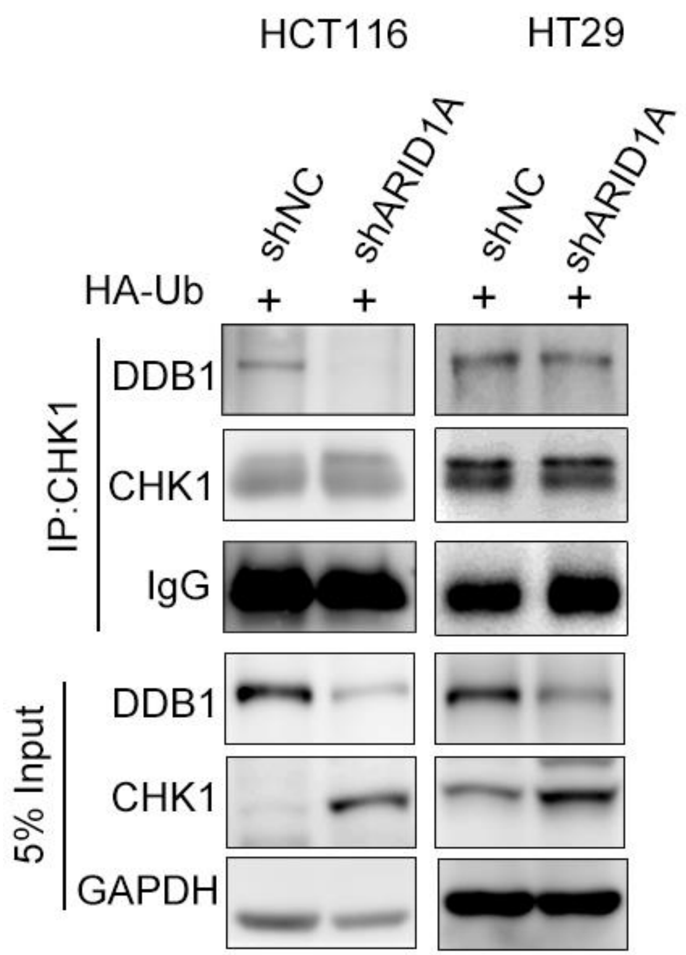
HCT116
HT29
shNC
shARID1A
shNC
shARID1A
HA-Ub
+
+
+
+
IP:CHK1
DDB1
CHK1
IgG
5% Input
DDB1
CHK1
GAPDH

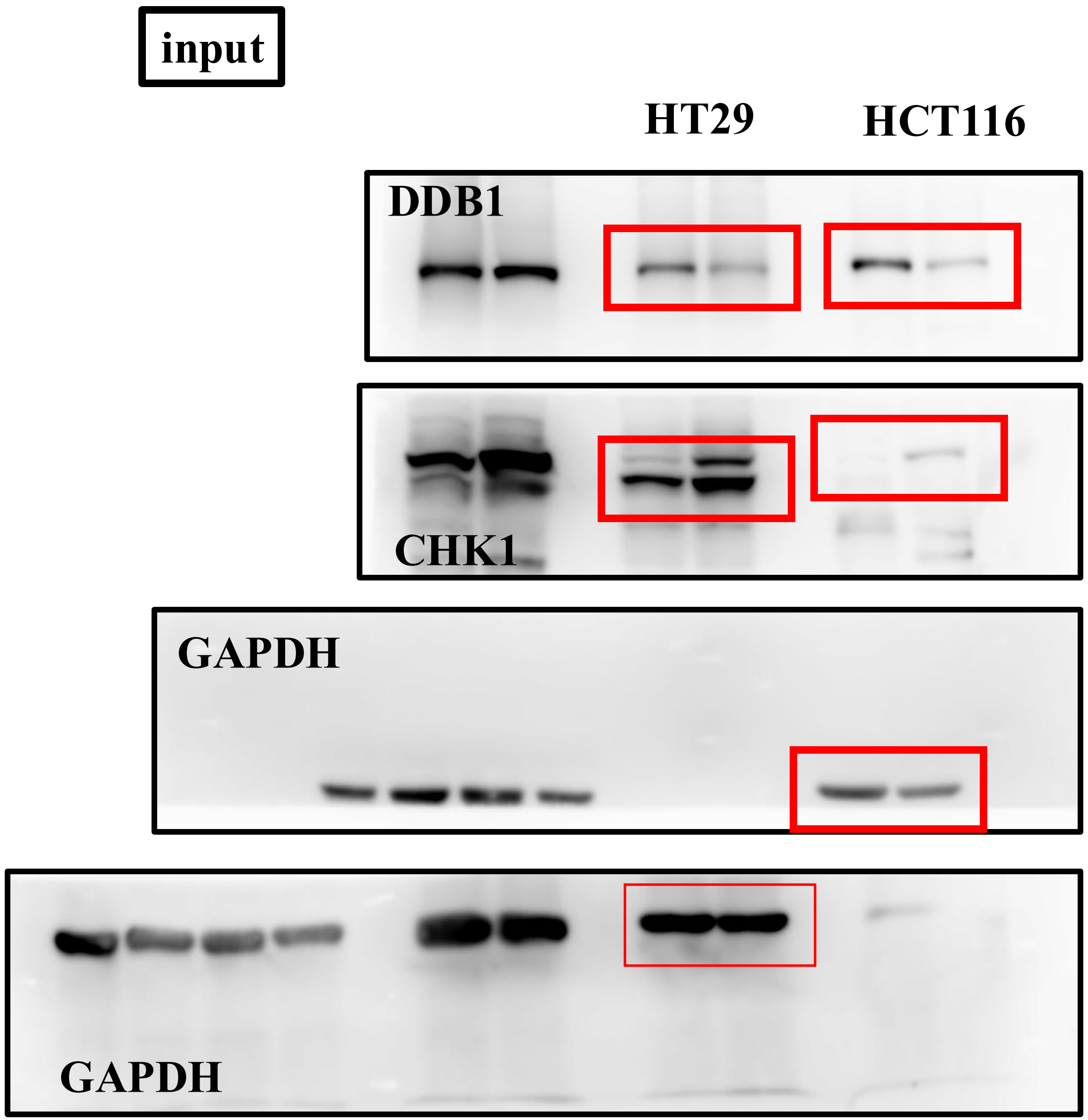
input
HT29
HCT116
DDB1
CHK1
GAPDH
GAPDH

Figure 4C

C

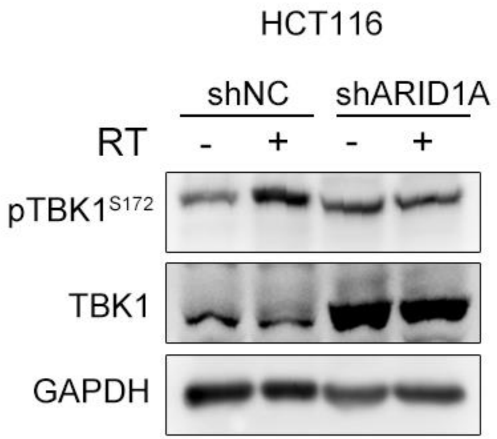
HCT116
shNC
shARID1A
RT
-
+
-
+
pTBK1S172
TBK1
GAPDH

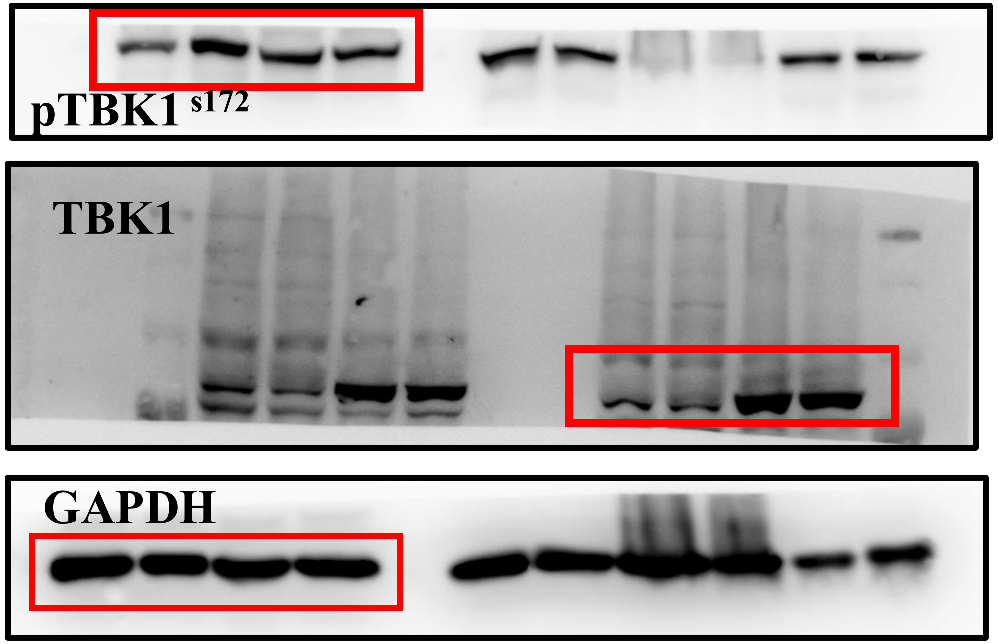
pTBK1 s172
TBK1
GAPDH

Figure 4D

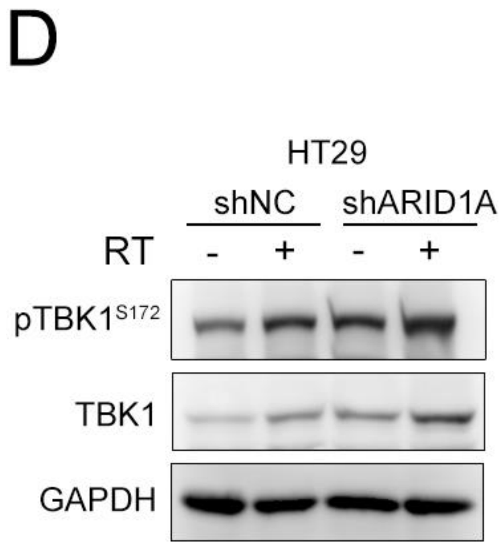
D
HT29
shNC
shARID1A
RT
-
+
-
+
pTBK1S172
TBK1
GAPDH

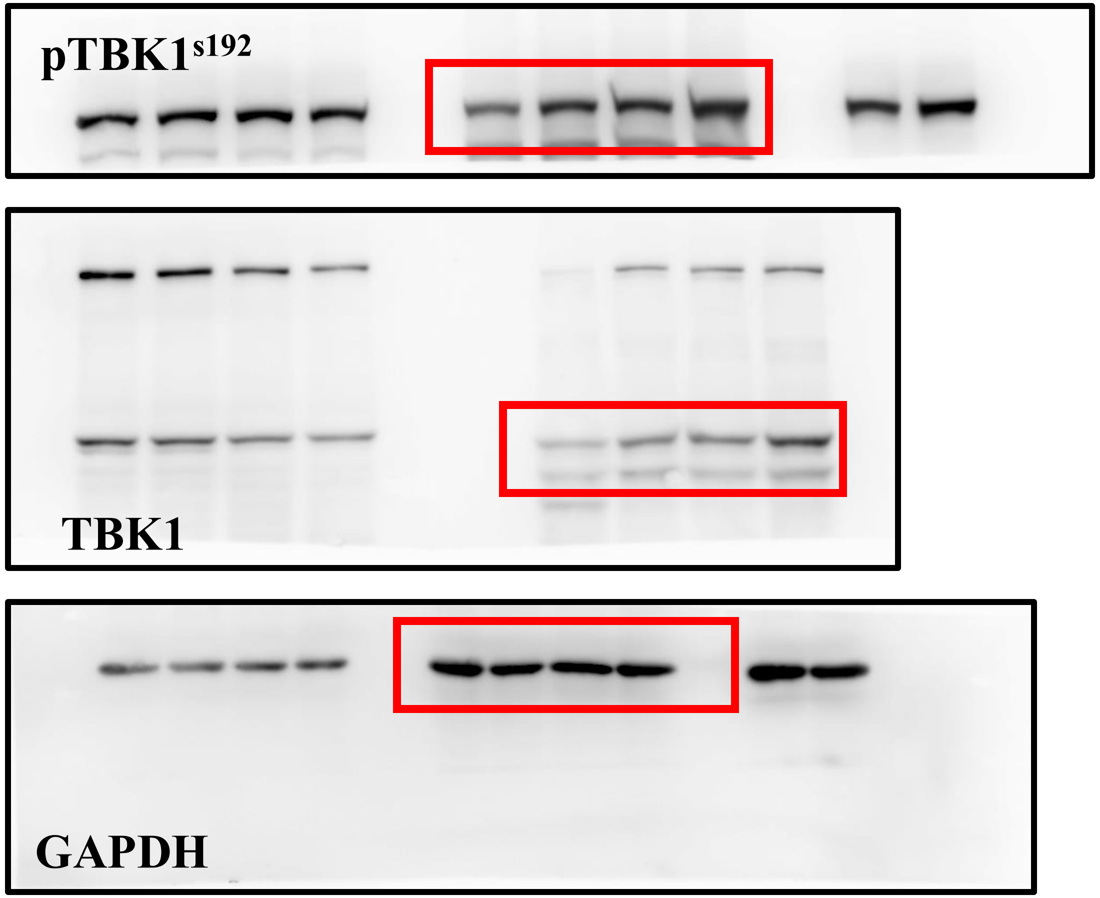
pTBK1s192
TBK1
GAPDH

Figure 6F

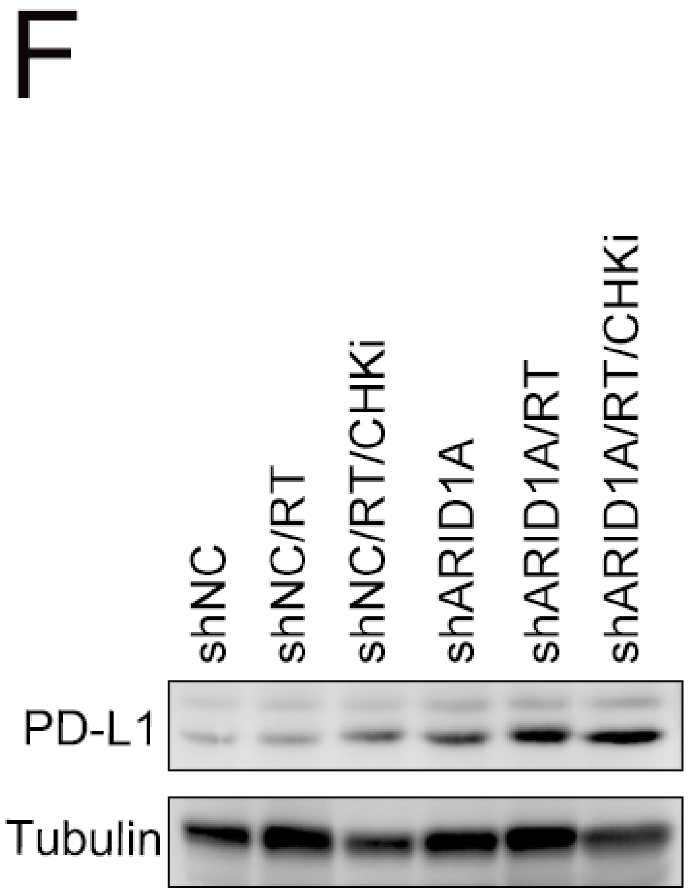
F
shNC
shNC/RT
shNC/RT/CHKi
shARID1A
shARID1A/RT
shARID1A/RT/CHKi
PD-L1
Tubulin

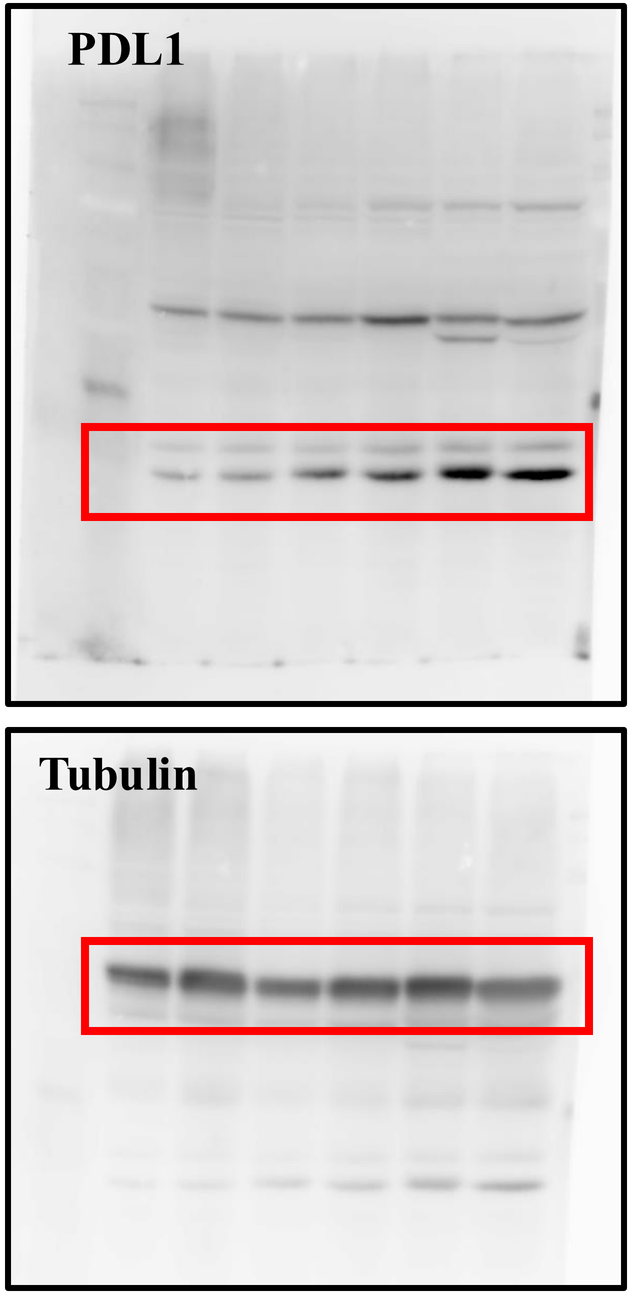
PDL1
Tubulin

Supl. Figure 2

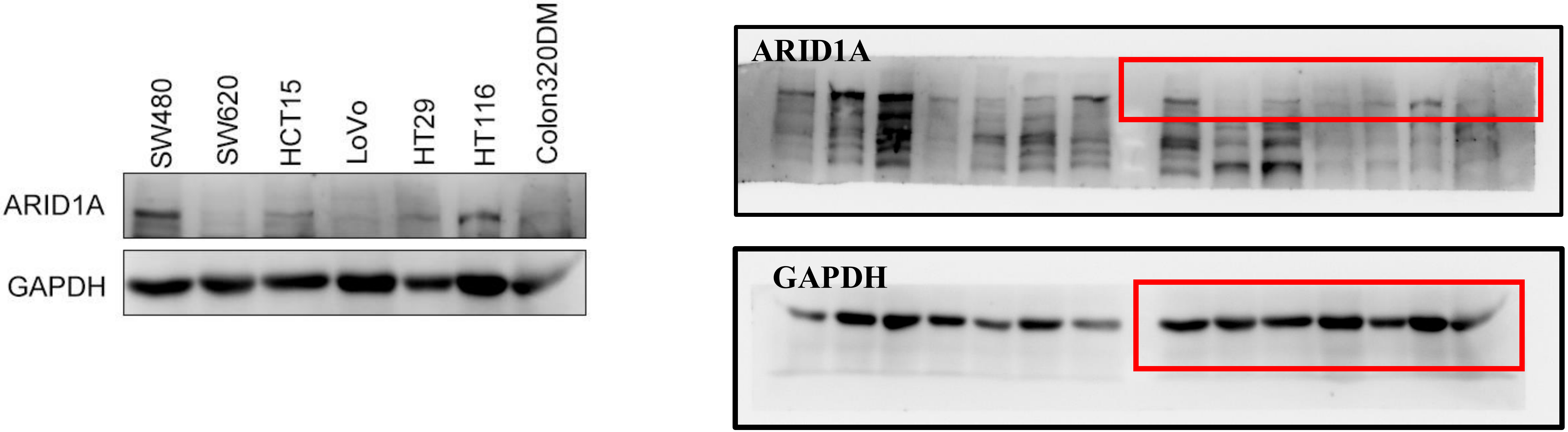

Supplement: Supplementary file 2 — Raw data from western blot [file 41419_2025_7912_MOESM2_ESM.pdf]
